# Exposure modelling in Europe: how to pave the road for the future as part of the European Exposure Science Strategy 2020–2030

**DOI:** 10.1038/s41370-022-00455-4

**Published:** 2022-08-02

**Authors:** Urs Schlüter, Jessica Meyer, Andreas Ahrens, Francesca Borghi, Frédéric Clerc, Christiaan Delmaar, Antonio Di Guardo, Tatsiana Dudzina, Peter Fantke, Wouter Fransman, Stefan Hahn, Henri Heussen, Christian Jung, Joonas Koivisto, Dorothea Koppisch, Alicia Paini, Nenad Savic, Andrea Spinazzè, Maryam Zare Jeddi, Natalie von Goetz

**Affiliations:** 1 Federal Institute for Occupational Safety and Health (BAuA), Friedrich-Henkel-Weg 1-25, D-44149 Dortmund, Germany; 2 Exposure and Supply Chain Unit, European Chemicals Agency (ECHA), P.O. Box 400, FI-00121 Helsinki, Finland; 3 Department of Science and High Technology, University of Insubria, 22100 Como, Italy; 4 National Institute for Research and Safety (INRS), Pollutants Metrology Division, Nancy, France; 5 National Institute for Public Health and the Environment (RIVM), Antonie van Leeuwenhoeklaan 9, 3721 MA Bilthoven, The Netherlands; 6 Exxon Mobil Petroleum and Chemical B.V., Hermeslaan 2, 1831 Machelen, Belgium; 7 Quantitative Sustainability Assessment, Department of Environmental and Resource Engineering, Technical University of Denmark, Produktionstorvet 424, 2800 Kgs Lyngby, Denmark; 8 TNO, Department Risk Analysis for Products in Development, P.O. Box 80015, 3508 TA Utrecht, The Netherlands; 9 Fraunhofer Institute for Toxicology and Experimental Medicine (ITEM), Nikolai-Fuchs-Strasse 1, 30625 Hannover, Germany; 10 Cosanta BV, Stationsplein Noord-Oost 202, 1117 CJ Schiphol-Oost, The Netherlands; 11 German Federal Institute for Risk Assessment (BfR), Max-Dohrn-Str. 8-10, D-10589 Berlin, Germany; 12 Institute for Atmospheric and Earth System Research (INAR), University of Helsinki, PL 64, FI-00014 UHEL, Helsinki, Finland; 13 Section 1.3 Exposure Monitoring—MGU, Institute for Occupational Safety and Health of the German Social Accident Insurance (IFA), Alte Heerstr. 111, 53757 Sankt Augustin, Germany; 14 European Commission Joint Research Centre (JRC), Ispra, Italy; 15 Center for Primary Care and Public Health, Unisanté, Route de la Corniche 2, 1066 Epalinges, Switzerland; 16 Swiss Federal Institute of Technology (ETH Zurich), Rämistrasse 101, 8092 Zurich, Switzerland; 17 Swiss Federal Office of Public Health (FOPH), Schwarzenburgstrasse 157, 3003 Bern, Switzerland

**Keywords:** Exposure models, European Exposure Science Strategy, ISES Europe

## Abstract

Exposure models are essential in almost all relevant contexts for exposure science. To address the numerous challenges and gaps that exist, exposure modelling is one of the priority areas of the European Exposure Science Strategy developed by the European Chapter of the International Society of Exposure Science (ISES Europe). A strategy was developed for the priority area of exposure modelling in Europe with four strategic objectives. These objectives are (1) improvement of models and tools, (2) development of new methodologies and support for understudied fields, (3) improvement of model use and (4) regulatory needs for modelling. In a bottom-up approach, exposure modellers from different European countries and institutions who are active in the fields of occupational, population and environmental exposure science pooled their expertise under the umbrella of the ISES Europe Working Group on exposure models. This working group assessed the state-of-the-art of exposure modelling in Europe by developing an inventory of exposure models used in Europe and reviewing the existing literature on pitfalls for exposure modelling, in order to identify crucial modelling-related strategy elements. Decisive actions were defined for ISES Europe stakeholders, including collecting available models and accompanying information in a living document curated and published by ISES Europe, as well as a long-term goal of developing a best-practices handbook. Alongside these actions, recommendations were developed and addressed to stakeholders outside of ISES Europe. Four strategic objectives were identified with an associated action plan and roadmap for the implementation of the European Exposure Science Strategy for exposure modelling. This strategic plan will foster a common understanding of modelling-related methodology, terminology and future research in Europe, and have a broader impact on strategic considerations globally.

## Introduction

In exposure science, computational models have essential roles in extrapolating, estimating, generalising, complementing and sometimes even replacing measurements. Many exposure metrics are not accessible by measurements, either because they cannot be measured directly (e.g. intake fraction, short duration), are not accessible due to ethical issues and compliance with human-subjects protocols or because the effort of measuring them would be enormous. It is acknowledged that models cannot, and should not, replace the collection of good quality exposure measurements [1], where accessible. Models and experimental data are closely linked: mathematical models are designed based on mechanistic understanding of exposure that stems from existing experimental data, and then evaluated with different data sets (existing or new) to explore their applicability domain.

Following the definition of “exposure science” by the Europe Regional Chapter of the International Society of Exposure Science (ISES Europe is one of several regional chapters of the International Society of Exposure Science, a scientific society working on all aspects of exposure) [2], the strategy of ISES Europe on exposure models encompasses models for human and environmental exposure. In these models, the “receptor” can either be the human being or environmental compartments. Additionally, in specific models (e.g. bioaccumulation) the receptor can be a selected species. Despite fundamental differences in the modelling approach for humans and environmental exposures, large parts of the methodology and related issues are similar for all receptors and areas (e.g. the modelling of partitioning based on substance properties). A common strategy for exposure modelling in Europe can address issues in human and environmental exposure modelling at the same time, while encompassing differences.

In Europe, exposure modelling has considerable momentum due to parts of the European chemical legislation that base decisions on risk assessment, rather than hazard assessment (e.g. the Biocidal Products Regulation EC 528/2012, the Plant Protection Products Regulation EC 1107/2009 and the REACH regulation EC 1907/2006), fostering a growing need for exposure science [3–5].

Under REACH, most identified uses of chemicals have no available measurements, thus, exposure and regulatory risk assessment in Europe largely rely on models [6, 7], or [8]. Furthermore, the recently published European Chemicals Strategy [9] and the goal of “Circular Economy” (for a general introduction to the topic see https://ec.europa.eu/eurostat/web/circular-economy) emphasise the reorganisation of substance assessment in Europe (e.g. by addressing mixtures and harmonisation), which creates the need to further develop modelling approaches.

Based on these considerations, ISES Europe identified exposure modelling as a priority area. The exposure science modelling working group (WG) was mandated to assess the current challenges and future needs for (chemical) exposure modelling in Europe. This paper presents the strategic objectives, actions and recommendations regarding exposure modelling in support of the European Exposure Science Strategy 2020–2030. The main focus of the whole strategy is on exposure assessment of chemicals, but the scope will be enlarged in the future to other stressors, e.g. noise, light and biological agents.

## Methods

### General approach and process

The strategy for exposure modelling in Europe was developed based on several rounds of expert consultations via three workshops in 2018, 2019 and 2020, as laid out in Fantke et al. [2].The ISES Europe WG on exposure models assembles a broad expertise in the different areas of exposure modelling (occupational, consumer and general population, environmental) and gathers experts from different European countries and institutions comprising academia, regulators, consultants, and industry.

The status quo regarding exposure modelling was assessed by collecting and reviewing the most relevant and most used models and tools for exposure assessment. The main emphasis was on models used in Europe, since some aspects of exposure modelling (e.g. exposure factors, regulatory requirements in occupational settings) are not readily transferrable across the globe. To tease out areas of possible improvements, the WG first identified and grouped gaps and requirements. Based on this, goals were defined and prioritised according to urgency and feasibility. Finally, important stakeholders were identified together with strategic objectives and associated actions where ISES Europe could act as facilitator. This led to a roadmap and recommendations for exposure modelling within the European Strategy for Exposure Science.

### Definitions

#### Models and tools

Exposure models for chemical substances aim at assessing substance levels at a specific point in time, in a specific medium. They consist of a set of mathematical equations with model parameters, and mostly are coded for fast computing of exposure estimates. In order to make their use more practical, models are often integrated into tools with a user-friendly interface. The terms “model” and “tool” can be distinguished as follows:

- An exposure model (here “model”) is a conceptual or mathematical representation of the exposure process [10, 11]. As such it encompasses a concept, set of input parameters and mathematical equations that are defined based on a dataset or another source of past experience regarding exposure phenomena.
- A modelling tool (here “tool”) is an embodiment of one or more exposure models within a standalone or web-based software. An individual exposure model may be integrated in different tools.

Models can rely on describing mathematically the physical principles that determine the transfer of the substance from its source to a receptor (mechanistic models), but also on the use of historical data (empirical models) and probability distributions (stochastic models), as well as expert judgement. Deterministic models aim to yield the same outcome for a given scenario, while stochastic models also include random effects. Some models also combine two or more of these principles, and various models and model types also include different elements of variability and uncertainty. The models can also include the fate of substances by describing the processes for degradation, reaction and partitioning. Depending on the aim of the exposure model, they can vary applicability domain, level of detail, and type of the output result.

#### Model evaluation

The quality of models depends mainly on their ability to reliably represent real exposure levels and/or the level of conservatism. Various concepts and theories have been developed for assessing the predictive ability of models, as well as different names that have been proposed for different stages [12, 13]. In order to use the most comprehensive term, we summarise all tests regarding predictive ability and reliability under “model evaluation”. This comprises testing against external data, comparison with other models, analysis of between-user variability and the assessment of documentation quality (“operational analysis” [14]), or internal plausibility checks.

#### Standard exposure scenario

In the context of exposure modelling, a standard exposure scenario is defined as “a combination of facts, assumptions, and inferences that define a discrete situation where potential exposures may occur…” [10, 11]. Exposure estimates depend decisively on the scenario, and since an unlimited number of different scenarios is imaginable, some regulatory settings (e.g. for the environmental fate of pesticides [15]) have defined standard exposure scenarios. These scenarios were constructed as conservative representatives for the variability of real settings and are requested for regulatory applications. Apart from ensuring a conservative assessment, standard scenarios allow a better comparison of different models.

### ISES Europe exposure model inventory

Developing an overview of existing models/tools is essential, to identify knowledge gaps. In Europe, there is no official inventory of models available. However, models have been reviewed and recommended by different regulatory bodies [16–18] and projects like the OMEGA-NET project [19]. Based on these compilations, and the expertise of the WG members, the ISES Europe model inventory was created. An overview of the models, along with basic descriptions of the models was created (see Table 1 for an overview of information collected for each model and supplementary material for the inventory, which includes references for each model). Although the inventory cannot be fully completed, the aim was to include the most important exposure models and tools used in Europe at the present time. The ISES Europe model inventory is intended to be a living document that is publicly accessible via the ISES Europe platform on the ISES Europe Website (https://ises-europe.org/exposure-platform/data-and-information-sharing, go to ISES Europe Exposure Model Inventory) and curated by the ISES Europe WG Exposure Models. Model developers and model users can contact the WG via the ISES Europe homepage.

**Table 1:** Information for models and tools collected in the ISES Europe Model inventory.

Descriptor (Column header)	Explanation of the descriptor
Short name	Short name of a model/tool, typically used for identifying a tool, in many cases abbreviations
Name of the model/tool	Full name of the model/tool
Exposure target	The human (sub)population (consumer, worker, general population) or environment (compartment)
Route of exposure	Route of exposure for humans (inhalation, dermal, oral) or environmental compartment (water, soil, air)
Sources of exposure	Activity or material where the substance is released or emitted and leads to exposure
Product class/chemicals/substances	Type of chemical (substance, mixture…), product class (cosmetic, pesticide…), form (vapour, particle…)
Tier/complexity	Classification of use of the tool regarding complexity, and if applicable characterisation into tiers used in regulatory exposure assessment (tier 1-screening tool, tier 2-more complex, tier 3)
Strengths	Specific strengths identified by the tool/model owners and/or during this analysis
Limitations	Specific limitations identified by the model owners and/or during this analysis
Evaluation status	Level of evaluation the model/tool achieved by the owner and/or independent research, number of evaluation studies, type evaluations that were performed…
Source/reference/download	Homepage/publication
Platform	e.g. Excel spreadsheet, Windows-based executable, Java Desktop Application…
Availability	e.g. Free, registration necessary, commercial…
Level of maintenance	Information if the model/tool is maintained or if it is—after the first development/publication—unchanged
Owner/developer	Institute/company that developed the model/tool or makes it available now
Language	Language used in the model/tool
Model input	Character of the inputs needed for the model/tool, e.g. value bands, qualitative expressions, quantitative values
Model structure	Short description of the theoretical background of the model/tool, e.g. Differential equations based on physical-chemical laws, quantitative values, distributions, semi-quantitative control banding…
Model output	Description of the output the model/tool produces, e.g. value bands, distinct values, qualitative assessment…
Tool	Yes/no if this is considered a tool
Model	Yes/no if this is considered a model
Remarks on model/tool	Short remarks about the model/tool that were identified during this analysis
Version available	Version number of the model/tool that is available right now
Last update	Year of the last update of the model/tool
Edited by	Name of the ISES Europe Expert who edited this entry of the inventory

The model descriptors are selected and ordered to allow exposure assessors a fast overview of available models, as well as highlighting their most important properties. For further information and in depth detail on the models, the respective websites and references should be consulted.

The exposure models chosen for the inventory vary in terms of theoretical background and applicability domain, reflecting their different use contexts and scopes. Models or tools that had no information available on the internet (homepage, publication) were excluded from the inventory. The inventory also does not include models designed for toxicological evaluations. However, dosimetry- or PBPK-models are included in the “Dosimetry & PBPK” Table, as these describe internal exposures and therefore, can be considered as exposure assessment models or tools. In order to make the inventory more transparent, it is divided into different tables that address different receptors, but all of them follow the structure as shown in Table 1. Models/tools that are meant for occupational exposure assessment are listed in the table “Worker”. The table “General population” combines exposure models or tools for consumer exposure and environmental exposure of humans. Exposure or emission models/tools for environmental compartments or organisms are listed in the table “Environmental Exp. (ecosystem)”.

Within the tables, the models/tools are in alphabetical order. It was not possible to fill in each of the cells, since for some models/tools the necessary information is not publicly available. However, the inventory will be continually updated to help assist exposure assessors in their selection of models/tools.

## Results

### Status quo and needs for exposure modelling in Europe

The status quo of the exposure model/tool landscape in Europe was captured in a model inventory (SI). The needs for the next 10 years were clustered under four general themes that are further detailed below (original SWOT analyses see Supplementary Material), with special focus on the role of the ISES Europe model WG.

#### Improvement of existing models/tools

Exposure models/tools are available for both generic exposure assessments in different sectors and for answering specific questions in explicit sectors [20]. However, the predictive ability of exposure models is still often debated by experimental scientists, as the evaluation status differs largely among models [21]. Furthermore, in contrast to the established quality controls for experimental measurements, quality plans are not commonly defined and applied to exposure models or tools. Similarly, data for defining model parameters are often not available, thus limiting the efficient and reliable application of models [22, 23].

Independent evaluation of models is an essential process in building confidence among users in their abilities [23]. The created model inventory showed that the evaluation status of most models/ tools is unclear. Albeit, conducting a complete evaluation of a model/tool, that is checked against independent measurement values, over its whole application domain may be prohibitively costly. However, most models could be, at least partly, evaluated through peer review [23], by reviewing the documentation status and the usability testing of the software (conceptual evaluation and operational analysis) [13]. The model WG could facilitate a peer review process or, in cooperation with the respective model/tool developers, provide guidance for self-review and documentation (e.g. developing evaluation criteria).

Next to the mathematical description of physical processes, models rely on data. Therefore, for improving models, a central element is to ensure access to existing data and to advise on data collection. Ideally, all data should be made available in an open database that would include all the exposure data collected/measured over the last few decades.

Improvement of models is also needed regarding specific processes. One example is spray application, e.g. hand held spraying for surface coating or application of disinfectants, but also the application of plant protection products (PPP): spraying results in a direct release of substances into the air, so that they are available for human inhalation and deposition on the skin. The deposition of aerosols in the lungs is size dependent [24], so that exposure depends on the size distribution. Size of the aerosols in turn depends on the spraying device and many other factors, such as size reduction of droplets due to evaporation of volatile components, containment and spraying nozzle. In practice, accurate information on these parameters is often not available, because well documented spray exposure measurements are scarce [25]. Further research is required on new modelling approaches such as relative source strength [26], harmonisation between models regarding input parameters and identification of standard spray scenarios.

In environmental modelling, scenarios and spray situation descriptions are generally available for the spray drift of PPP. The knowledge of spray drift at application time is very relevant in ecosystem exposure assessment, as spray drift can directly be attributed to the substantial amount of PPPs in the aquatic ecosystems. A modelling tool (Spray Drift Calculator) was therefore developed by the Joint Research Centre to calculate the drift, so that appropriate mitigation measures can be recommended [27].

#### Development of new methodologies and support for understudied research fields

With the rise of big data technology and omics measurements, a wealth of data becomes available that stays useless if not structured [28]. To use this data efficiently, new modelling strategies need to be further developed, e.g. Bayesian approaches to integrate data and model [29, 30]. In the new EU partnership on chemical risk assessment PARC [31] starting in 2022, data sharing is considered a key element and it is planned to develop common databases for Europe. Furthermore, the partnership intends to work on aggregate exposure and enhancing models for regulatory risk assessment.

Much of the new data that becomes available relates to information on personal time-activity tracking. Since exposure is strongly dependent on the exposure scenario and place of exposure, such data is immensely useful for exposure modelling. New approaches to use this data include agent-based modelling [32], which could be the basis for more realistic exposure modelling for air pollutants such as PM10.

Further, mathematical modelling and statistical methods should be applied for read-across of exposure data [33]. For most chemicals, monitoring and exposure data are not available [8]. Although, if chemicals have similar structural similarities and properties this data could be derived from other chemicals by read-across. For assessing chemical properties, Quantitative Structure-Property-relationships (QSPR)-methods are used. Thus, by combining QSPRs with monitoring databases, a full framework for read-across can be established [34]. Recent approaches also include the construction of quantitative structure use relationships for screening [35].

Below are specific areas where new developments are considered most urgent.

##### Dermal exposure

Both in occupational and consumer exposure settings, data and modelling approaches for dermal exposure are underrepresented compared to inhalation exposure. There are some tools available (e.g. RISKOFDERM, MEASE, ECETOC TRA, Crème cosmetics, PACEM), all of which require a number of input parameters that are often not available or difficult to obtain (e.g. the dermal absorption). In addition, only very limited information regarding the evaluation of dermal exposure models is available [36]. Limitations also exist regarding modelling approaches for processes, such as incidental exposure by splashes, contact with contaminated surfaces, deposition of airborne substances on the skin [37] and subsequent uptake into different skin layers [22].

However, recent efforts have been made to advance dermal exposure modelling. Based on measurements of dermal exposure at workplaces, McNally et al. [38] concluded that hand exposures resulting from a particular task were each dominated by one or two of the mass transfer processes identified in Schneider et al. [39]. This is promising and could lead to more mechanistic approaches for dermal models.

##### Aggregate exposure

Aggregate exposure summarises exposure to several or all known exposure sources for one single substance [40], based on which a full human risk assessment can be conducted [41]. The EU legislations that cover items with regular exposure (e.g. food, cosmetics) mandate aggregate exposure assessments as part of the safety assessment of ingredients or contaminants. However, a comprehensive aggregate assessment remains a challenge because there are not fit-for-purpose tools available. For example, the ISES Europe model inventory does not include models that can be used to calculate aggregate exposure estimates for workers. Generic frameworks for aggregate exposure assessment have been developed (e.g. Merlin-EXPO—https://merlin-expo.eu/, SHEDS—https://www.epa.gov/chemical-research/stochastic-human-exposure-and-dose-simulation-sheds-estimate-human-exposure), but their applicability has only been demonstrated in a small number of case studies [42–44]) and require harmonisation regarding parameter choice, scenarios, etc.

The principle need for aggregate exposure assessments may be addressed by advancing person-oriented approaches/frameworks and generating freely accessible lists of products uses and occurrence for chemicals. Additionally, high-quality data for environmental and consumer exposure levels are needed for distinct sub-populations (e.g. workers or vulnerable sub-groups).

##### Combined exposure (to mixtures of substances)

A methodology that is suitable for mixture exposure and risk assessment is still under development. Due to the very large number of possible combinations of chemicals, identifying priority mixture scenarios represents a particular scientific and regulatory challenge [45]. Recent EU-funded research projects tested some approaches for mixture identification (e.g. EDCMixRisk—https://edcmixrisk.ki.se/, EuroMix—https://www.euromixproject.eu) from chemical monitoring and assessed probabilistic co-exposure modelling data for the environment (e.g. SOLUTIONS [46, 47]). Despite this progress, significant knowledge and data gaps still remain with regard to human exposure and toxicity data, and environmental exposure and ecotoxicology [48], respectively.

Generally, co-exposures can be anticipated because of the co-presence of multiple substances within a formulated product, co-use or co-presence in the environment. In the past, co-exposures have been investigated using human biomonitoring and environmental monitoring data [49–51]. For example, Tornero-Velez et al. [52] found that pesticide co-occurrence in the child care centres was not random but highly structured, leading to the co-occurrence of specific pesticide combinations. However, the scope of such analyses is restricted to the chemicals included in the monitoring programmes based on concerns regarding hazard, and hence does not cover all “real-life co-exposures”. Complementary exposure modelling thus is necessary to account for substances that are not monitored or monitored infrequently. Ideally, what is needed for the assessment of combined exposure is the ability to predict the likelihood of specific co-exposures, so that the most likely co-exposures can be considered in a cumulative risk assessment. For exposure through diet, some methodologies have recently been developed and applied [53, 54]. But for most substances, and their sources, data is lacking on occurrence that prohibits similar approaches. Creating occurrence databases and advancing mixture modelling therefore is crucial, as well as co-exposure to physical nuisances [55, 56].

##### Exposure to objects and solid materials

Substances emitted from objects and solid materials pose specific challenges regarding exposure assessment. Typically, a transport phase within the material (diffusion) and a mass transfer from the material surface to the skin, mouth or into air characterise emission for these materials. Models to estimate emission and exposure to substances in articles and solid materials have been proposed [57, 58] and implemented in tools (ConsExpo—https://www.rivm.nl/en/consexpo, CEM—https://www.epa.gov/tsca-screening-tools/cem-consumer-exposure-model-download-and-install-instructions, DustEx—https://www.dustex.nl/, reviewed by Eichler et al. [37]. However, large gaps exist in the availability of appropriate input data for these models, e.g. specifically for diffusion and partition coefficients. Those depend on both the substance and the material, and may vary by multiple orders of magnitude between different substance/material combinations. Brandsch et al. [59] proposed a predictive model for migration related parameters for food packaging materials. Others [60–62] applied molecular dynamics simulation methods to model diffusion coefficients in-silico, a method that could also be applicable to estimate other model parameters. Despite their usefulness, these approaches cover only a limited portion of the possible substance/material combinations and therefore need to be complemented by modelling approaches for other applicability domains (e.g. non-food polymers, inorganics and polymer additives).

##### Challenges regarding specific groups of substances

The WHO chemical safety programme has identified ten chemicals or groups of chemicals of major health concern (https://www.who.int/teams/environment-climate-change-and-health/chemical-safety-and-health/health-impacts/chemicals), which include several metals. Since metals undergo speciation in the environment and in biota, they are especially difficult to model. In addition, various metal complexes and organic substances are being used in nanoparticulate form that changes size and agglomeration status once chemical or physical conditions change. Finally, inorganic substances, such as inorganic cyanides, are particularly difficult to assess due to complex environmental reaction kinetics and dynamics [63].

However, there are many more organic substances and substance classes that pose special challenges, which have not yet been addressed satisfactorily by exposure modelling. To name a few, per- and polyfluoroalkyl substances (PFASs), polar/ionising substances and substances of unknown composition (unknown or variable composition, complex reaction products or biological materials (UVCB)) are mentioned. For PFASs, conventional fate and exposure modelling approaches based on octanol-water partitioning are not applicable, requiring the production of additional test data and partitioning approaches for PFASs [64]. A recent review of environmental fate and exposure models highlights that obtaining reliable property data is a problem for polar and ionising substances [65]. These chemicals are a large fraction among those registered under REACH [66], but only few approaches exist for estimating their partitioning and bioaccumulation properties [67–70]. Substances of unknown composition (UVCBs) are also challenging, because classical chemical fate modelling relies on substance properties, which per se are not available for UVCBs. For them, statistical approaches or probabilistic models, processing ranges of substance properties, may provide solutions.

#### Improvement of model use (the human factor)

While it is generally recognised that training on workplace exposure measurements or toxicological assessments is important, there is no similar training requirements for exposure modelling. This absence of quality assurance in exposure modelling seems inconsistent with the high-quality that is expected for risk assessments. Several studies have shown that this lack of quality assurance leads to a high inter-user variability, not only for complex expert models [71], but also for simpler “Tier1”-models [72, 73]. Lamb et al. stresses that training on the use of models and the interpretation of the results should be an essential part of education courses in exposure assessment, which ideally should be certified. Another possibility is the use of a consensus/team approach (analogy to round-robin tests for analytical methods), where models are selected and importantly have a group of assessors (instead of one person) that calculates an exposure estimate and an average estimate or consensus value is used [71, 73, 74].

The observed high inter-user variability can further be reduced by making the models and tools more user friendly, by providing online help and an interactive staffed helpdesk.

Standard exposure scenarios for occupational settings would further improve the comparability of assessments: exposure modellers must translate information about a workplace situation into model parameters and describe the exposure using a range of determinants. This involves a number of decisions that render the assessment subjective [21]. Agreeing on a set of standard scenarios would help to remove some parts of this subjectivity and resulting variability in the models. Standard exposure scenarios have already been implemented in some environmental exposure tools (e.g. the generic regional model in EUSES [75–77], CHESAR [78]) and the scenarios used for pesticides in groundwater and surface water as implemented in the FOCUS models [15]. The FOCUS scenarios were identified by expert groups, comprising of the regulatory, academia and industry sectors, in a consensus process and form that is now the backbone of pesticide environmental risk assessment by ensuring comparability among substances (https://esdac.jrc.ec.europa.eu/projects/surface-water). Similarly, for consumer exposure first tier standard scenarios have been developed for ECETOC-TRA and default values are available for ConsExpo that help to standardise the assessments.

#### Regulatory needs for exposure modelling

Under European legislation, exposure assessment is under the full responsibility of industry, but authorities can evaluate the assessments. The rigour of these evaluations depends on the respective regulation. In all European registration and approval frameworks, exposure assessments aim to determine the conditions under which a substance can safely be used. Complementary to this, authorities determine priorities for regulatory assessments that compare options for regulatory risk management or demonstrate the need for restrictions. For both purposes, the models applied are largely the same, but e.g. different parameterisation are used.

The overarching requirements for exposure models/tools used in regulatory contexts can be summarised in a few basic points:

- Applicability domain and the limitations of the model should be clearly described and available.
- Definitions and impact of input parameters should be very transparent.
- Complexity should be limited to enable robust reproducible assessments.
- Models should fit into a tiered assessment approach.
- Models should enable generic safety assessments for broader categories of uses and use conditions.
- Translation of the model input parameter values into conditions of use (e.g. risk management measures) should be attempted.

Where exposure assessments are an integral part of regulation (e.g. environment, general population and consumers for industrial chemicals and biocides), some tools have been specifically recommended by European authorities. For example, EUSES is now the default model in CHESAR for first tier environmental assessments of industrial chemicals and biocides, and MACRO, PELMO, PRZM-GW, and PEARL are used for first tier groundwater modelling of pesticides [79]. This harmonisation is helpful for ensuring consistency and equal treatment in the regulatory process, but less advanced in occupational exposure assessment. As a result, various tools for occupational exposure assessment are in use that sometimes provide conflicting results [8]. Even among authorities, there is no consensus to date on how to agree on scientifically sound tools/models for regulatory workplace exposure modelling.

### Vision for exposure modelling

Based on the aforementioned needs analysis, a vision for exposure modelling for the future has been developed by the WG. The following elements are key to advance the science and to support decision-making in the area of chemical exposure modelling:

1. Exposure models are well documented, ideally long-term supported (maintained, helpdesk) by a hosting body, and at times updated to reflect major scientific progress. Quality assurance planning and a process for implementing a quality assurance plan is implemented in the development of new methodologies [80].
2. Exposure models are evaluated independently against data or other models to understand and best focus their applicability ranges and performances (e.g. by sensitivity and uncertainty analysis). The results of such exercises are used for improvement of the models.
3. Exposure modellers have a background in natural or computational sciences and are well trained by additional post-graduate training modules or a dedicated master’s programme. They follow widely agreed best practices and guidance where available.
4. Exposure modellers engage with other relevant stakeholders (including regulators) from the start when developing a model.
5. Best practices for exposure modelling (e.g. documented scenario and parameter selection, incorporation of uncertainty, etc.) are compiled, readily available and accepted by all relevant stakeholders.
6. Exposure models used in the regulatory context are fit for purpose for the specific regulation and accepted by the relevant regulatory body.
7. The integration of exposure models along the whole path from source to dose is advanced, as well as the integration of models with data, where appropriate.
8. Understudied fields of research in exposure modelling receive adequate attention and funding.

### ISES Europe Strategy for exposure modelling

Based on the needs identified in “Status quo and needs for exposure modelling in Europe” section and the vision in “Vision for exposure modelling”, a set of different strategic objectives and associated actions have been identified. These are summarised in Table 2 and further explained below.

**Table 2:** Strategic objectives SO (grey) for exposure modelling (based on identified needs) and associated actions and recommendations.

	Requirements to achieve SO	Actions ISES Europe, short/mid term	Actions ISES Europe, long-term	Stakeholders apart from ISES Europe	Recommendations to other stakeholders
Improvement of existing models and tools (SO-1)					
Ensure adequate model documentation	Common understanding about adequate documentation	Development of a minimum standard for model documentation	Development of a handbook on best practices that includes guidance for adequate documentation	Model developers, standard setting bodies	Funding agencies/Model developers: Ensure that projects for model development include also documentation and open access to the model code
Ensure model maintenance and hosting (e.g. coverage of IT-platform costs)	Maintenance, further development, business model etc. need to be part of project plans for model development	Encourage discussions between scientists and regulators regarding solutions		Model developers, Standard setting bodies (?), regulatory authorities, tool users Funding agencies	Regulatory bodies: develop financing models for long-term maintenance of models Industry: invest, as maintenance of models is the basis for maintenance of registration
Address uncertainties (model uncertainty, parameter uncertainty, scenario uncertainty etc.)	Model/tool development for uncertainty assessment		Highlight uncertainty assessment in the best practice handbook	Model developers, scientific community	Scientists/Regulators: define uncertainty factors for default values, mathematical simplifications, and assess other sources of uncertainty in models
Evaluate model parameters	Evaluation of model parameters (e.g. activity pattern, vapour pressure) and how they are included in models	Provide a platform for the discussion among scientists		Model developers, scientific community	Model developers: define applicability domains/ranges for parameters regarding chemical properties; investigate and display probability distributions for model parameter values e.g. across similar workplaces or consumer uses
Evaluate models	Agreed methodology Data (with an agreed minimum standard) for evaluation against external measurements	Develop criteria for the evaluation of models (e.g. with respect to model acceptance, applicability, scientific robustness), including standard statistical parameters for the comparison of model results and measurements	Develop standard scenarios for testing of models Develop criteria for the reliability of model estimates (e.g. scoring system) Expert assessment of models (peer review)	Model developers, Scientific community	Model developers: define applicability domains for models where lacking
Enhance the compatibility of models (e.g. linking external and internal exposure, simultaneous assessment of different exposure pathways)		Enhance dialogue between disciplines Create a forum for interdisciplinary project development		Scientific community	
Evaluation of parameters missing in models (e.g. turbulence)	Some processes are currently neglected in all standard models, effect often unclear → research need	Develop a list of probably relevant parameters	Encourage the exchange between model developers and regulatory bodies	Model developers, Exposure scientists, research funders	Exposure scientists: scientific projects Regulatory bodies: check if models contain all relevant parameters for compliance testing Research funding bodies: launching of research programmes for modelling
Development of new methodologies, support for understudied research fields (SO-2)					
Development of read-across strategies using models	Agreed methodology Data (with an agreed minimum standard) for read-across	Develop criteria for high-quality data, that can be used for read-across		Model developers, Exposure scientists, research funders	Exposure scientists: scientific projects
Use of Data	Access to relevant databases Compatibility of databases (e.g. by using a common identifier) Harmonisation of data dictionaries and quality criteria of data	Cooperation with WG Data, promote FAIR principle		Model developers, Exposure scientists, research funders Data owners	Exposure scientists: scientific projects
Identify exposure situations where models/tools are missing or existing models are known to be “inappropriate” and develop new modelling approaches	Common understanding about the question for which situations additional models are necessary			Model developers, Exposure scientists	Model developers: e.g. develop/integrate new equations for polar and dissociated chemicals Develop model for dermal exposure assessment
Improvement of model use: the human factor (SO-3)					
Addressing the large inter-user variability of models	Standardisation of model use Awareness of model applicability domain, strengths and weaknesses Adequate education, certified trainings Improvement of user friendliness, online help in the tools, interactive staffed helpdesk Agreed methodology for “difficult situations”	Publicly available (e.g. on the website of ISES Europe) information about training courses, webinars, e-learning, instruction movies, manuals on models/tools. Provide an overview of available models and respective information Adaption and use of usability testing and usability inspection for exposure models	Guidance on model choice and use (best practices handbook), including peer review of existing models/tools; make publicly available and visible Better Education → cross-link to WG EDU Establishing a two-person-rule or consensus/team protocols when using models	Universities, Exposure scientists, Model or tool developers, Regulatory bodies	Define applicability domains were lacking Tool developers: taking into account human-computer interaction science for improved user friendliness of models Create a sustainable platform to translate and provide the models in the local languages Regulatory bodies: Define quality criteria for tools
Define modelling standards including uncertainty assessment (e.g. in comparison with standards for measurements, EN 689)	Agreed minimum standards on how modelling should be documented, e.g. - Modeller - Description of scenario/situation - Used model/tool - Input parameters, incl. uncertainty - Statistical information - Conclusion, additional RMM - Uncertainty assessment…	Develop guidance on good modelling practice based on existing work, promote e.g. by ISES Europe web cross-link to WG data repositories because of data quality and WG Education		Exposure scientists	Universities: plan courses for model development
Regulatory requirements for exposure modelling (SO-4)					
Evaluation and definition of scenarios for regulatory purposes See also above as a task for model evaluation	Agreed scenarios that are most relevant for regulatory purposes	Development of some (a small number of) examples how such scenarios could look like		Regulatory commissions (e.g. RAC?)	Regulatory bodies: define requirements for models, if they are used for regulatory purpose
Translation of model parameters into safe use-advice (Communication along the supply chain of components in modelled safe use scenarios that need to be observed for risk management measures)	Common understanding about how parameters of models or outputs can also be regarded as risk management measures (e.g. room size)	Build a “platform” to evaluate models for this purpose		Model developers, regulators	Model developers/industry/regulators: development of translation tables from parameters to use-advice
Targeted model development to support regulatory processes for product safety	Check if available models are also beneficial for regulatory purposes			Model developers, regulators, industry	Model developers: improve/amend existing models to make them fit for regulatory purpose
Need for acceptance and common framework for modelling-based occupational assessment	New development, maybe in line with situation in consumer and environmental modelling where broadly accepted and maintained models exist One component may be to promote the use of several models for comparison (TREXMO) Another component: Connect a broader range of tools (beyond ECETOC TRA) to Chemical Safety Assessment under REACH (via CHESAR)			Model developers, regulators, industry	Model developers and regulators: enhance communication

The scientific community assembled in ISES Europe can address many, but not all of the needs that are identified in “Status quo and needs for exposure modelling in Europe” section. ISES Europe as a society can bring together exposure scientists from different disciplines to stimulate the development of interdisciplinary projects, and agree on research needs and knowledge gaps to generate the basis for successful project applications. However, to ensure funding of such projects, stakeholders in regulation, research and research funding should get involved.

**Fig. 1:**
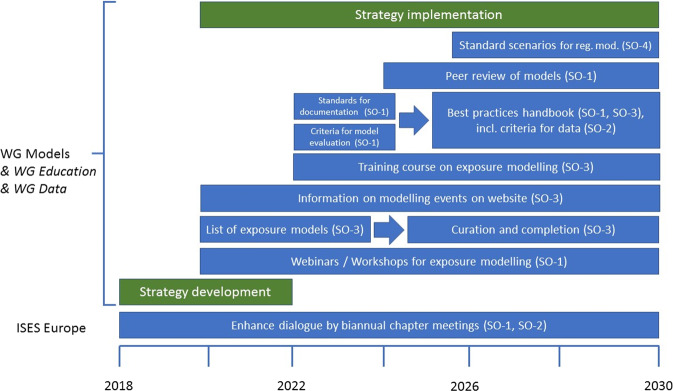
Roadmap for ISES Europe Actions from 2018–2030 in response to the strategic objectives (SO) for exposure models. The roadmap is described from bottom to top in green bars: strategic process, blue bars: action points for ISES Europe (different WG will cooperate for the action points).

Therefore, the strategy was subdivided into the core strategy for ISES Europe with strategic objectives and actions for the WG on models (see Fig. 1 and below), and recommendations to other stakeholders (section “Recommendations to stakeholders for advancing exposure modelling in Europe”).

#### Improvement of existing models and tools (SO-1)

Model evaluation and generation of measurement data (for model development and evaluation) are crucial for model improvement. ISES Europe should assist in organising the peer review of models or, in cooperation with the respective model/tool developers, provide guidance for self-review and documentation. For this, ISES Europe should bring together the general scientific community, model developers and the regulatory bodies for developing evaluation criteria for testing models and tools. To develop and implement the strategy, a model WG and a WG on databases were founded under the same umbrella with the possibility of joint meetings. Thus, ISES Europe will enhance exchange between experimental scientists and modellers on gaps in exposure data (e.g. by implementation of quality assurance in the development process of new methodologies [80] or by organising webinars/workshops for specific areas of debate [81]).

#### Development of new methodologies (SO-2)

The development of new methodologies is important in many aspects of exposure modelling. ISES Europe brings together exposure scientists from different disciplines to learn from each other and promote networking. However, this area is especially resource-intensive, so that effective funding mechanisms are crucial. With the new partnership on risk assessment, PARC [31], the EU commission has created a funding model that might also stimulate research on exposure in general. ISES Europe members actively intervened to strengthen the consideration of models and model parameters in this research partnership by active participation, and were partly successful. ISES Europe intends to serve as a promotor for alliances that are essential to influence decisions on funding, e.g. by continuing to organise its annual meetings as a mix between scientific conference and dedicated workshops of the WG.

Regarding aggregate exposure, the “one substance, one assessment strategy” adopted by the European Commission in October 2020 [9] provides incentives for harmonisation and policy coordination on the level of sectorial legislations. ISES Europe can help to develop a common understanding on basic elements of aggregate exposure models, and thus further stimulate research.

#### Improvement of model use (SO-3)

An important factor in improving the quality of model use is training and accessibility of information. In this respect, the WG Education and WG models cooperate to design a curriculum for exposure science that also includes a module on exposure modelling. The ISES Europe website has plans to list existing curricula and trainings. The website will also host a list of the models that are most frequently used in Europe. This list has been developed as a living document for reference. The current state of this list has been discussed in this paper and is included in the Supplementary Material.

ISES Europe should facilitate expert groups that develop a common understanding of conservative default values and scenarios for First Tier assessments. This shall be achieved by organising workshops, where tool developers, owners and users test different standard scenarios simultaneously using different models, so that a better understanding about the performance of conservative default values and scenarios is achieved.

#### Regulatory requirements for exposure modelling (SO-4)

For occupational exposure models, ISES Europe should start a process for agreeing on one platform and one actively managed and resourced process. This should be supported by authorities and industry with a vested interest in a commonly accepted set of models. In this process, ISES Europe will identify gaps in the current tools and priority development needs. A further step will be to develop standard scenarios for occupational settings for use in regulatory exposure assessments.

From this set of strategic objectives and proposed actions the following action plan and roadmap (Fig. 1) was developed for ISES Europe and the WG Models. Timing and selection of the different action points was based on needs, ease of implementation and the willingness of volunteers to start work on a specific field.

## Discussion

### General

The development of a European strategy for exposure science is a self-mandate of ISES Europe. As such, it represents the view of the experts involved in the society and in the respective WGs. However, since the WG on exposure models is open to everybody and a large number of recognised experts in the respective fields were involved in the process of developing and reviewing the strategy for exposure modelling, this strategic building block captures the perspectives of a large panel of European exposure scientists. Mirroring the current representations in ISES, more exposure scientists focused on human exposures are active in the WG, than environmental exposure scientists. Although, both in the WG and in the drafting group for the strategy, environmental exposure scientists were involved. A potential bias towards human exposures may have resulted, but the general conclusions hold for both human and environmental exposure modelling.

**Fig. 2:**
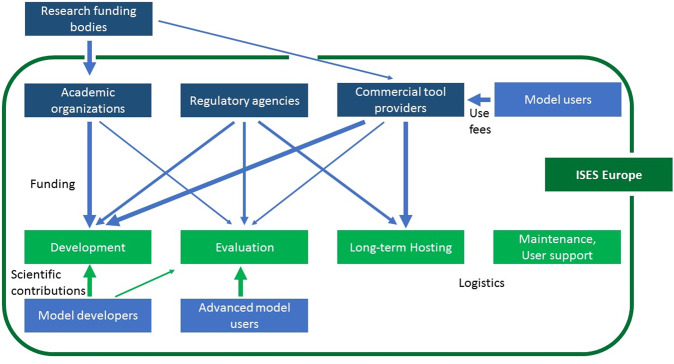
Relationships between stakeholders with a role for exposure models. Dark green box and arrows: proposed coordination role for ISES Europe, dark blue boxes: strategical stakeholders (funding), blue boxes: operational stakeholders, light green boxes: tasks along the life cycle of models, blue arrows: funding, green arrows: scientific contributions (the thickness of arrows shows the strength of the relationship).

An important component of the strategy building was to identify the role for ISES Europe in advancing exposure science and in the implementation of this strategy. It was concluded that it is not in the remit of ISES Europe to finance research on improving exposure models. Though, the chapter can serve as an independent platform and driver for exchange, bringing together scientists and practitioners from different institutions that together develop research projects and case studies, and apply for funding. Further, ISES Europe can promote exposure science with stakeholders and funding bodies involved in the life-cycle of exposure models (Fig. 2).

In Europe, most research funding for exposure model development comes from the European Commission and country-specific research funding bodies. Further, commercial organisations fund the development of models (e.g. the Crème models or Stoffenmanager®). Figure 2 illustrates the relationships between the different stakeholders (blue boxes) and where effective funding and support schemes are established for the necessary tasks (green boxes). Model development is often only one component in larger projects (e.g. aiming at health assessments and not model development per se) and many model developers are working on short-term projects in academia. Consequently, those models are not always hosted, and rarely evaluated by other users. Maintenance and support are practically non-existent for academic models. Regulatory agencies also play an important role in the development of models, since many regulations heavily rely on models. Nevertheless, even if regulatory agencies often host and evaluate models, funding these tasks is difficult for such organisations, and it is mostly not possible to provide user support.

A major shortcoming is the limited funding for evaluation and for hosting, maintenance and support of non-commercial models, which mainly occurs as no established procedures are defined for funding these logistical needs. For commercial models, hosting, maintenance and support are provided by the company, but out of fear of being copied by competitors, often commercial models are less transparent than other models (e.g. source code are not open access), which is a major disadvantage for model evaluation.

Therefore, a key challenge is that all tasks required for good modelling practice are not regularly funded. There is no identified body in Europe that has the obligation to host and maintain models. Since representatives of all stakeholders are present in the WG models, ISES Europe may take the lead to develop a sustainable framework for funding and supporting important exposure models, which will be selected in line with the needs identified in “Status quo and needs for exposure modelling in Europe” section.

The identified research needs have to be communicated and taken up by funding agencies and research organisations.

### First steps towards actions identified for ISES Europe

For some of the actions presented in Fig. 1, work has already started and concepts were developed for implementation. These are presented in the following.

#### Development of guidance on best-practices

Many identified needs relate to the lack of guidance for exposure modelling. Some regulatory bodies attempt to give recommendations on models (e.g. ECHA, R.14 [8]) and some publications are available on best practices in environmental exposure modelling [65, 82, 83], but an agreed guidance on best practices is missing. A guidance would enhance the reliability of and the trust in exposure modelling. In order to develop best practice documents, already available guidance documents need to be reviewed. This review will then serve as a basis to identify guidance documents that can have an overarching relevance for several frameworks (regulatory and scientific). In a next step, relevant information (from several evaluated guidance documents) will be combined into one guidance document and reviewed in a public consultation, to improve the document and reach a common understanding. In order to achieve regulatory acceptance, a joint publication of the guidance with relevant regulatory bodies should be envisaged. ISES Europe could facilitate this process by organising the development, discussion and agreement of the guidance. Further, training material, models/tools lists and guidance will be assembled by ISES Europe.

#### Development of evaluation criteria for exposure models

A medium-term goal identified in the strategy is to improve the acceptance and applicability of exposure models. In this context, the ISES Europe WG defined actions regarding the development of a model inventory, and the definition of evaluation criteria. Such criteria are ideally addressed for all models in the model inventory.

Internal model evaluation criteria are related to the model concept (e.g. internal consistency, robustness, underlying theory and assumptions for the model purpose), the consideration of uncertainties, and the model applicability domain. Furthermore, an operational analysis that comprises a check of inter-user variability and test of the user-friendliness and model accessibility as applied elsewhere is necessary [84]. For the latter, established methods (e.g. usability testing and inspection) [85, 86] could be adapted to the scope of exposure modelling. Additionally, external evaluation criteria are necessary, e.g. comparison of model estimates with measured data [21]. This step often requires transformation of model estimates, or experimental results, to a common metric for comparison [87, 88], along with other adaptations, as the critical appraisal of the different statistical parameters used is necessary. This includes defining appropriate quality criteria for the measurement data, and is therefore an area for cooperation with the ISES Europe WG on data repositories.

Model evaluation should be conducted, for example, under the auspices of an independent expert panel, whose members are free of competing interests. On these grounds, some tool owners have already established a supervising board (e.g. ConsExpo and Stoffenmanager® [89]), but for many models, such an independent expert panel still needs to be defined.

#### A commented model inventory

The quality of exposure modelling is influenced not only by the model, but also by the model use. Hence, a lack of quality in exposure modelling may be caused by the user not being aware of the available models, their features and applicability domains. In order to facilitate a good overview on models, the ISES Europe WG on exposure models initiated the creation of the ISES Europe model inventory, introduced in section “ISES Europe exposure model inventory”. This is planned to be a living document hosted on the ISES Europe website, which may later be developed into an online database.

As a final aim, we intend to summarise a tiered set of exposure models [90] for the assessment of exposure situations. This will be further developed into a decision matrix for selecting models for a specific decision context in a tiered approach.

#### The ISES Europe Model WG as a platform for scientific exchange for exposure modellers

With computational power growing, the integration of models for human and environmental exposure assessment is becoming achievable. At the same time, developers of such integrated models need to understand the concepts used by the different sectors and create suitable interfaces. This knowledge can be provided within the ISES Europe WG on exposure models. The members of this WG agreed to work on the short- to medium-term actions identified in this strategy paper, but also value the WG as a platform to share knowledge, identify interdisciplinary projects and create common understandings of processes and modelling approaches.

In a virtual workshop on the “Theoretical Background of Occupational Exposure Models” [91], the participants recently discussed the needs regarding the enhancement of occupational exposure models. Some activities were identified that would improve the status quo, which will enable potential synergies and collaboration with other relevant scientific bodies (e.g. the OMEGA-NET project (https://occupationalexposuretools.net/) or the OECD working party on exposure assessment (WPEA, https://www.oecd.org/chemicalsafety/risk-assessment/oecdactivitiesonexposureassessment.htm)).

### Recommendations to stakeholders for advancing exposure modelling in Europe

An important goal for exposure modelling is to establish better funding mechanisms for model evaluation, hosting and support (see section “General”). In this respect, ISES Europe can serve as a facilitator of discussions by approaching the relevant organisations and decision makers involved.

With the new Green Deal [92] and the EU chemicals strategy for sustainability [9] towards a toxic-free environment, chemical risk assessment has gained more attention in the European Union. However, exposure assessment is still under-recognised in the field of risk assessment when compared to effects assessment. Similarly, exposure modelling is often undervalued in comparison to measurement data such as human biomonitoring and environmental monitoring, but essential for prospective assessments and source identification. For the risk assessment of the future, exposure modelling will have to complement exposure measurements.

In order to advance exposure modelling in Europe, stakeholders have to be addressed with specific recommendations that together feed into common goals. For this purpose, goals have been identified (see Table 2) and the following subsections illustrate how the different stakeholders can orchestrate their actions in the pursuit of these goals. In addition to the operational stakeholders identified in Fig. 1 (model users, model developers, tool providers), regulatory decision makers and the scientific community in general are addressed.

The scientific community for exposure modelling (and exposure science as a whole) develops, evaluates and uses exposure models as part of scientific research. There are two main groups of scientists involved in exposure modelling: model developers who develop, assess and use models, and models users who use existing models. Model developers may be independent researchers but also regulatory institutions or industry-funded organisations. Model users may be from academia (e.g. health/environment effects research), from government/regulatory bodies (e.g. risk assessment and management) or from industry (e.g. fulfilment of regulatory obligations, risk assessments). The different roles and affiliations induce conflicts of interest that influence the discussion between the different stakeholders. The ISES Europe WG on exposure models provides a neutral podium to advance exposure modelling in Europe.

#### Recommendations for regulatory decision makers

Regulatory decisions in Europe are taken on different levels for different purposes. At the highest level, the European Commission and the Member States make decisions of far-reaching political importance. Agencies at EU-level (e.g. ECHA, EFSA) and country-level prepare these decisions by providing the necessary scientific background.

In many cases, current official documents and procedures show a lack of awareness of the importance and needs of exposure science in general and exposure modelling in particular. For example, the European Commission adopted in October 2020 the new EU’s Chemicals Strategy for Sustainability [9] mentions the role of exposure modelling only on an abstract level, while much more emphasis is given to monitoring. European regulatory bodies and national agencies therefore should thrive to integrate exposure science and modelling more prominently in their overarching strategies.

On an operational level, it is necessary that EU and country agencies to cooperate and align practices when using exposure modelling for identification of regulatory priorities, assessment activities or conclusions on medium level regulatory decisions. As an inspiring example, ECHA’s Chemical Safety Assessment tool for producers of chemicals CHESAR—https://chesar.echa.europa.eu/home, for human exposures at present only includes a TIER 1 exposure calculation engine based on industries’ ECETOC TRA tool. In the effort to advance CHESAR, ECHA established a stakeholder platform to support CHESAR development and involve exposure tool owners, industry and Member States in this activity. Further, since exposure models play an important role in transporting scientific evidence, decision makers need to get involved in the model development process.

With regard to the growing importance of aggregating and combining exposure, harmonisation of exposure assessment across different legislations is needed. This effort is supported by expert groups like e.g. the REACH Exposure Expert Group (REEG—https://echa.europa.eu/de/reach-exposure-expert-group), the Panel on Plant Protection Products and their Residues (PPR—https://www.efsa.europa.eu/en/panels/ppr) or the Panel on Contaminants in the Food Chain (CONTAM—https://www.efsa.europa.eu/en/panels/contam), but concrete action points towards harmonisation are not yet defined.

Entirely different decisions are made by research funding bodies on the resources that are allocated to research activities on exposure assessment. For example, the proposed European Partnership under Horizon Europe “Partnership for the Assessment of Risk from Chemicals” (PARC [31]) will open up the opportunity to assign more funds to exposure science, including the advancement for exposure modelling in Europe.

#### Recommendations for the scientific community

Most of the relevant exposure assessment models and tools that are currently used in Europe have come to age and are based on methods and data that are partly outdated. Only few model developers regularly update and maintain their models and tools, but this is crucial to advance exposure modelling. Up-to-date models enhance trust by better model predictions and cost-effectiveness by computational efficiency. Multidisciplinary research, state of the art scientific methodology and digital innovations foster advanced tools, methods and models. The analysis and use of current and available data can improve existing models and tools or initiate new developments for models and tools in areas where they are missing.

In addition to the improvement of existing or the development of missing models and tools, further needs have been identified above. These primarily address the need for harmonisation and independent evaluation of models and tools but also modelling parameters. For example, a database may be beneficial. This database should include source parameters for different equipment, devices and techniques. Additionally, an independent platform could include agreed default parameters for specific scenarios from different regulatory frameworks. Currently, several bodies exist in parallel that could serve as an independent platform. These different bodies have a different focus on exposure modelling and none have a really overarching claim, as most of them focus on specific regulatory frameworks (e.g. REACH, PPP) or protection goals (e.g. environment, occupational health and safety).

#### Recommendations for model developers and tool providers

For model developers it is important to be aware of the current knowledge gaps, such as aggregate exposure assessment, exposure to mixtures or to objects and solid materials.

Crucial in the use of models/tools is good documentation. Crucial points to consider when developing or improving tools are to enhance user friendliness, to implement help systems and to provide guidance for choosing suitable input parameters [93]. When a new tool is developed, provision of model updates should be part of the project plan and business model. Guidance on parameter choice is specifically important for parameters that have more qualitative definitions (e.g. use categorisation, intrinsic dustiness, type of setting, risk management measures), because for these there is a large inter-user variability of parameter choices that has been observed [73].

Model and tool developers have an important role in the education of model users. For example, the development and establishment of fit-for-purpose training courses for exposure assessments are only possible if developers and authorities co-operate.

Furthermore, in order to enhance trust and understanding by decision makers, when developing or using a specific modelling approach a clear policy question is key, alongside clear communication of data, uncertainties and the background of the model. To ensure these qualities, the involvement of decision makers is critical when developing models for regulatory purposes.

#### Recommendations for model users

There is a responsibility of model/tool users to make sure that they are competent to perform exposure modelling that is of sufficient quality. In response, the ISES Europe strategy includes strategy elements on education in exposure science [94] and the development of a set of good practices for exposure modelling. Furthermore, round robin tests comparing user performance could reveal pitfalls that, when remediated, would reduce the inter-user-variability [73, 95, 96]. Obviously, user education and the development and implementation of good practices needs a joint effort of several parties (see Fig. 2): single companies or authorities will not succeed when acting alone. Tool users, industry associations, regulators and exposure scientists (e.g., tool developers, university) need to agree on the basis of high-quality exposure modelling. The involvement of the scientific community (i.e., relevant scientific societies) as well as an independent body for quality assurance is obligatory.

As a first step, the ISES Europe will provide a list of available courses on exposure modelling that is not exhaustive, but will be updated regularly (https://ises-europe.org/exposure-platform, go to “Exposure science training courses”).

### Advancing exposure modelling in an international context

The most prominent and recent strategy document in the area of exposure science is the US National Research Council Report “Exposure Science in the 21st Century” [28]. It dedicates a paragraph in the summary to explaining where “models and information-management tools” are important and how they should be improved. Emphasis is placed on the development of integrative tools and approaches that treat an agent from source to receptor and take into account multiple stressors, as well as include uncertainty assessment. Further, it stresses that with the advancement of observational data in various fields (e.g. big data in geo-statistics or for consumer behaviour). It is also essential that the capacity to analyse this data and to integrate data from different fields is strengthened.

Owing to the international nature of the field of exposure modelling, these general research needs vocalised by US experts are very similar to those identified here. However, differences are more pronounced when it comes to concrete steps proposed to realise the vision, since exposure modelling can only reasonably be applied in the respective legal framework. International cooperation should therefore focus on the general scientific research needs that are put forward in international WGs such as the OECD WPEA, WHO International Programme on Chemical Safety) and in cooperation between scientists in ISES on a global level.

## Conclusions

Exposure models are essential to improve the understanding of exposure for risk assessment and other purposes. In a bottom-up approach, exposure modellers in Europe from different areas of exposure science have agreed on four main strategic objectives with an associated action plan and roadmap for the implementation of the ISES Europe strategy for exposure science in the priority area of exposure models. These strategic objectives comprise improvement of existing models/tools, development of new methodologies, improvement of model use and regulatory requirements for exposure models. As a first step, based on already available compilations, and the expertise of the WG members, the ISES Europe model inventory was created. An overview of the models, along with basic descriptions of the models was created. Although the inventory cannot be fully completed, it can serve as reference for both exposure scientists and model users as it includes the most important exposure models and tools used in Europe at the present time. The ISES Europe model inventory is intended to be a living document that is publicly accessible via the ISES Europe platform on the ISES Europe Website. The scientists involved in the ISES Europe WG on exposure models intend to collaborate in the future to implement the strategy and to foster a common understanding of methodology, terminology and future areas of research. In the spirit of this common goal, the WG on models is open to all interested collaborators.

## Supplementary information

Reporting Checklist

Suplemtary Material
