# Supplementary material for: Exposure modelling in Europe: how to pave the road for the future as part of the European Exposure Science Strategy 2020–2030
Source: J Expo Sci Environ Epidemiol. 2022 Aug 2;32(4):499–512. doi: 10.1038/s41370-022-00455-4 (PMC9349043; doi:10.1038/s41370-022-00455-4)
Supplement: Supplementary file 2 — Suplemtary Material [file 41370_2022_455_MOESM2_ESM.pdf]

Read me

This model and tool inventory was assembled and is curated by the Working Group Exposure Models of the European chapter of the International Society on Exposure Science (ISES Europe).

Version number / Date: 1.0 / March 2022

This inventory cannot be complete but aims to include the most important exposure models and tools used in the EU now. The ISES Europe model inventory is intended as a living document that is accessible via the ISES Europe platform on the ISES Europe Website.

This table consists of four sheets (and this "Read me" sheet):
- Worker with tools and models for exposure assessment of occupational exposure due to handling of chemicals at workplaces

- General population (human) with tools and models for exposure assessment of humans via the environment and for exposure assessment due to handling of chemicals by consumers (including different sub-populations)

- Environmental Exp. (ecosystem) with tools and models for exposure assessment of environmental compartments due to emissions to the environment

- Dosimetry & PBPK with tools and models for “internal exposure assessment”

Models and tools that were specifically designed for the exposure assessment of nano materials are placed in one of the above-mentioned sheets. Most of those tools are developed for occupational exposure assessment and are placed in the worker sheet.

If a model or tool cannot be assigned clearly to one sheet only, it will be explained in detail in one of the sheets. In the other sheets there will be a reference to that sheet.

Models or tools that cannot be found in the internet (homepage of the model or tool or a scientific publication as a minimum) will not be included in this inventory.

If you, as a user of this inventory, find a mistake or want to add an important model, please get in contact with urs.schlueter@ises-europe.org.

short name	name of the model / tool	exposure target	route of exposure	sources of exposure	product class / chemicals / substances	tier / complexity	strengths	limitations	evaluation status	source / reference / download	platform	availability	level of maintenance	owner / developer	language	model input	model structure	model output	tool	model	remarks on model / tool	version available	last update	edited by
**ADL AMEM**	see sheet "General population (human)"	Different Targets	inhalation, dermal		articles																			
**ANSES tool**	ANSES Control Banding Tool for Nanomaterials	Worker		activities with nanomaterials	Solids, liquids, powders, aerosols.	screening tool	The tool has five hazard bands (HB1-5) and four emission potential bands (EP1-4) which are combined to give five control classes (control bands) (CL1-5). Scaling of the emission potential (EP1-4) is made according to the physical form of the nanomaterial at the beginning of the process.		unknown	https://www.anses.fr/en/system/files/AP2008sa0407RaEN.pdf	paper	free		ANSES	english	bands	control banding		no	yes			2010	US
**AOEM**	Agricultural Operator Exposure Model (AOEM), BfR	Worker	inhalation, dermal, oral	activities with pesticides, typical scenarios including the mixing/loading and the application of plant protection products	Plant protection products, pesticides	Can be used for a tiered approach: Tier I scenario corresponds to exposure considering no PPE but one layer of work clothes covering torso, arms and legs; Tier II scenario dermal and/or inhalation PPE factors can be chosen if [illegible]	Model for prediction of exposure of professional operators applying plant protection products outdoors, exposure mainly depends on the total amount of active substance used per day and is further described by additional factors or particular sub-scenarios.	Few data exist in the database for knapsack mixing/loading and for hand-held application in low crops. Due to the limited data no statistical model could be derived from them. In addition, data are completely lacking for high crop applications with knapsack sprayers and for low crop applications [illegible]	unknown	https://mobil.bfr.bund.de/cm/350/joint-development-of-a-new-agricultural-operator-exposure-model.pdf	paper	free	active	BfR	englisch	quantitative values	regression analysis, the underlying equations are based on log linear models for prediction of the 75th percentile and consist of exposure factors that were selected after a statistical analysis	several percentiles	no	yes	using previously unpublished field data collected between 1994 and 2009	-	2016	JM

**ART**	ART - Advanced Reach Tool	Worker	inhalation	activities with chemicals	Vapor and particulates	Tier 2	On-screen guidance helpful to understand the inputs, Bayesian update of the predictions using analogous data from the exposure data library or by uploading own data, helpful guidance on creating the data format to upload.	Cannot assess fumes, fibers, gases and dust from hot metallurgical processes. Not intended for consumer exposures. Can only be used for inhalation exposures.	several validation studies + peer-review studies to evaluate the validity (reliability) of the exposure estimates	https://www.advancedreachtool.com/	web-based	free (registration is needed)	active (V.1.5)	ART is jointly funded by HSE, Dutch Government, AFSSET, CEFIC LRI, Shell, Eurometaux, BOHS and GSK.	englisch	qualitative expressions, quantitative values, value bands	modifying factors with subsequent calibration, additionally use of physico-chemical relations, Bayesian modelling	several percentiles, Advanced statistics	yes	yes		1.5	2014	JM
**BAMA Indoor Air model**	BAMA Indoor Air model	Worker	inhalation		aerosol					https://www.bama.co.uk/product.php?product_id=11		available free of charge on BAMA website.		BRE Group	englisch									
**BROWSE model**	BROWSE (Bystanders, Residents, Operators and Workers Exposure models for plant protection products) model	Worker, general population	inhalation, dermal, oral	activities with plant protection products	Plant protection products, pesticides		can be used to calculate exposures for a range of pesticide applications. This browse software is designed to be used in regulatory risk assessments of pesticides but it has not been approved or adopted by authorised regulators and they will not currently		THIS BROWSE SOFTWARE IS PUBLISHED FOR TESTING AND REVIEW PURPOSES ONLY.	https://secure.fera.defra.gov.uk/browse/software www.browseproject.eu; andy.hart@fera.gsi.gov.uk	Java version 7	free	complete	The BROWSE project is supported by the EU 7th Framework Programme, ref. 265307.	englisch				yes			ver. 5.3	2016	
**CAREX**	CARcinogen EXposure (CAREX)	Worker	inhalation, dermal	activities with carcinogens	chemical carcinogens					https://oem.bmj.com/content/72/1/64			active		englisch									

**CB Nanotool 2.0**	CB Nanotool 2.0	Worker	inhalation, dermal	activities with nanomaterials	Engineered nano materials		Created so that a non-expert can use it.	The rating scale is based on professional judgment.	Was tested against 5 operations that are running with controls established by experienced professionals, and the model recommendations matched actual practice very closely.	https://controlbanding.llnl.gov/download	Access			David M. Zalk, PhD, CIH - Deputy Team Leader at the Lawrence Livermore National Laboratory (LLNL); Samuel Y. Paik, PhD, CIH - technical lead for the Industrial Hygiene Group within the Worker Safety and Health [illegible]	englisch		Control Banding							
**Contam**	Contam	Different Targets predominately industrial hygiene. Applicable also for consumer exposure assessment.	inhalation	activities with chemicals	vapour, particles	Tier 1 to 3 (depends on parametrisation)	CONTAM is a multizone indoor air quality and ventilation analysis computer program Built from a standard modeling approach (2-zone inhalation exposure model published in AIHA's modeling	CONTAM Library: https://www.nist.gov/el/energy-and-environment-division-73200/nist-multizone-modeling/software/contam/input-data	Validated (e.g. Jayjock et al. 2011; Abattan et al. 2020)	https://www.nist.gov/el/energy-and-environment-division-73200/nist-multizone-modeling/software/contam	Application	free	active	AIHA's Exposure Assessment Strategies Committee (EASC)	English	quantitative values, distributions	Differential equations based on physical-chemical laws	Quantitative values	yes	yes		3.4.0.0	2020	JK
**COSHH Essentials**	COSHH Essentials : Control of Substances Hazardous to Health	worker	inhalation	activities with chemicals	chemicals	screening tool	COSHH essentials is a simple tool based on an empirical approach to risk assessment and risk management.		some validation peer-review studies to explore the conservatism of the exposure estimates	https://www.hse.gov.uk/coshh/essentials/index.htm	web-based	free	active	developed in Great Britain by UKhealth safety excutive	englisch	qualitative expressions, quantitative values	qualitative control banding	exposure bands common benchmark of good practice for chemical users, manufacturers, suppliers and importers, as well as regulators and health professionals	yes	yes	COSHH essentials has proved to be a popular tool for communicating good control practice. It has attracted over 1 million visits to its site since its launch.			JM

**CropLife OPEX Tool**	CropLife OPEX Tool	Worker	inhalation, dermal	activities with plant protection products	Pesticides					http://www.fao.org/pesticide-registration-toolkit/registration-tools/assessment-methods/method-detail/en/c/1187029/	excel		complete	FAO - Food and Agriculture Organization of the United Nations	englisch						ainly based on the US EPA Occupational Pesticide Handler Exposure Data. Certain scenarios for handheld application have been used from the German Model.			
**dART**	Dermal ART	Worker	Dermal	activities with chemicals	chemicals					Goede, H. A., et al. (2019). Ann Work Expo Health 63(6): 624-636. 10.1093/annweh/wxy106				ART consortium	english	qualitative expressions, quantitative values, value bands	modifying factors and subsequent calibration, Bayesian modelling	several percentiles, Advanced statistics	beta version on Diamonds platform	yes				JM
**EASY-TRA**	EASY-Targeted Risk Assessment	Worker	inhalation, dermal, oral	activities with chemicals	several	screening assessments, conservative but multi-tier.		Europe		http://www.easytra.com/		licensed			englisch				yes	no	Parts of EASY TRA are based on ECETOC TRA, but EASY TRA includes a number deviations considerably changing the exposure value (e.g. linear instead of sublinear exposure reduction with time and concentration)			JM

**ECETOC TRA**	ECETOC TRA	Worker (also modules for consumers and environment available)	inhalation, dermal, oral	activities with chemicals	liquid and solid chemicals	Tier 1, conservative, worst case	ECETOC TRA is a widely applicable tool. The dermal estimation process is more crude than that for inhalation. Moreover ECETOC TRA is based on the descriptor system used in REACH (ECHA, 2010a) which provides a detailed and pragmatic approach of describing processes or tasks occurring at workplaces.	Gases are out of the scope of model, although ECETOC gives some additional advice how to deal with volatiles with vapour pressures above 30 kPa during the estimation of dermal exposure. ECETOC TRA is not directly applicable to molten solids (i.e. non-mineral solids) [illegible]	several validation studies, e.g. ETEAM + peer-review studies to explore the validity (reliability) of the exposure estimates	http://www.ecetoc.org/tools/targeted-risk-assessment-tra/	Excel spreadsheet version	free	active (V. 3.1)	ECETOC	englisch		initial exposure estimate and modification by factors		yes	yes	further development based on EASE	3.1	2014	JM
**EFSA calculator**	EFSA calculator	Worker	inhalation, dermal	activities with pesticides	Pesticides					https://www.efsa.europa.eu/en/efsajournal/pub/3874			complete	The European Food Safety Authority (EFSA)	englisch				yes	no	Agricultural Operator Exposure Model (AOEM) + US Pesticide Handlers Exposure Database (PHED)	Version 30 Mar 2015	2015	JM
**EMKG-Expo-Tool**	EMKG-Expo-Tool 2.0, the "Easy-to-use workplace control scheme for hazardous substances" (EMKG "Einfaches Maßnahmenkonzept für Gefahrstoffe")	Worker	inhalation	activities with chemicals	chemicals	screening	Only three input parameters, the tool's simple structure enables the user to distinguish quickly between critical and non-critical workplace situations by comparing with the substance specific DNEL. The tool offers a simplified approach to evaluate worker exposure and [illegible]	situations, where dusts are formed through abrasive techniques, open spray applications, the handling of gases, pesticides, operations giving rise to smoke (soldering, welding), operations giving rise to wood dusts, CMR substances	several validation studies, e.g. ETEAM	https://www.baua.de/EN/Topics/Work-design/Hazardous-substances/REACH-assessment-unit/pdf/User-Guide-EMKG-Expo-Tool.pdf	Java(TM) Desktop Application (MS Windows, Mac OS X, Linux) , therefore a Java Runtime Engine (JRE) must be installed	free	active	BAuA	englisch	value bands, qualitative expressions, quantitative values	semi-quantitative control banding, additionally use of physical-chemical relations for translation of input parameters	value bands	yes	yes	further development based on COSHH Essentials, needs improvements for several substance classes, e.g. dusty solids	2.0	2017	JM

**ENMs in research environments**	Control banding methodology to manage the safety of ENMs in research environments	Worker	inhalation, dermal	activities with nanomaterials	Engineered nano materials	Tier 1, 2 and 3	Created so that a non-expert can use it.	The rating scale is based on professional judgment.	Was tested against 5 operations that are running with controls established by experienced professionals, and the model recommendations matched actual practice very closely.	Groso A, Petri-Fink A, Rothen-Rutishauser B, Hofmann H, Meyer T. Engineered nanomaterials: toward effective safety management in research laboratories. J Nanobiotechnology. 2016;14:21. Published 2016 Mar 15. doi:10.1186/s12951-016-[illegible]		free	english	Groso et al.			control banding		no					
**EPA ExpoBox**	see sheet "General population (human)"	Different Targets, Overview platform	inhalation, dermal, oral	activities with chemicals			chemicals																	
**IEAT**	IEAT (Ingestion Exposure Assessment Tool)	Occupational	Dermal, ingestion		Multiple		ingestion exposure, one of very few models for ingestion			https://api.semanticscholar.org/CorpusID:38055762. linked to earlier work https://academic.oup.com/annweh/article/50/7/693/318136				IOM	English				no	yes				
**IH-MOD**	IH-MOD	Different Targets predominately industrial hygiene	inhalation	activities with chemicals	vapour, particles	Tier 1 to 3 (depends on parametrisation)	Built from a standard modeling approach (2-zone inhalation exposure model published in AIHA's modeling guide); probabilistic model; assesses long-term and acute exposures	Estimating some inputs may be difficult for novice users.	Validated (e.g. Jayjock et al. 2011; Abattan et al. 2020)	https://www.aiha.org/get-involved/VolunteerGroups/Pages/Exposure-Assessment-Strategies-Committee.aspx	Excel	free	active	AIHA's Exposure Assessment Strategies Committee (EASC)		quantitative values, distributions	Differential equations based on physical-chemical laws	Quantitative values	yes	yes		2	2020	JK

**iNano**	Indoor Exposure Model for dispersed aerosols and airborne nanoparticles	Occupational (indoor air and worker)	inhalation	activities with nanomaterials	aerosol, airborne dust, particles & nanoparticles	higher tier	Takes into account particles aggregation (especially for the nano range), particles surface deposition/sedimentation, source reduction by local controls and time dependance of all	Lacks a source library to facilitate the inputs when measurements are lacking.Does not deal with simultaneous multiple sources.	Validated using experimental test cases such as collected in Guichard, R., Tanière, A., Belut, E., & Rimbert, N. (2014). Simulation of nanoparticle coagulation under Brownian motion and turbulence in a differential–algebraic framework: [illegible]	Belut et al., 2019; DOI: 10.1111/ina.12579	Matlab based graphical user interface (GUI). Usable freely with Matlab runtime environnment.	on demand	active	INRS, France	English, French						alpha version, unknwown future			
**MEASE 2**	MEASE 2 ("the metals' EASE")	Worker	inhalation, dermal	activities with metals, inorganic substances and hot metalurgical processes	metals and inorganic substances	Tier 1, conservative, worst case	The use of MEASE 2 supports the user in selecting an applicable process category (PROC) from the recent list of PROCs given in ECHA guidance R.12. In addition, the user is able to select the relevant conditions of use from a large list of exposure determinants.	dermal exposure estimation is limited to hands and forearms with the exact exposed skin area depending on the PROC number	Based on MEASE 1 that was evaluated in ETEAM, + peer-review studies to explore the reliability of the exposure estimates	https://www.ebrc.de/tools/downloads.php	Windows 10 / Java 8	free (registration is needed)	active, continuous updates available	EBRC Consulting GmbH	English	quantitative values, value bands, qualitative expressions	initial exposure estimate and modification by factors	distinct values	yes	yes	further development based EASE expert system, from the TRA tool and from the health risk assessment guidance for metals (HERAG) the dermal part is coarser but relies on real workplace measurements and should [illegible]	Version 1.0 also MEASE 1 is still available on the EBRC homepage	2018	JM

**MIXIE**	A tool for the assessment of multiple exposure to chemical substances	worker	inhalation, dermal, ingestion	activities with chemical mixtures	chemicals		easy to use, almost no parameters needed	only the additive effects are used, other type of chemical interaction in body not taken into account (synergetic, antagonist, potentialization)		https://www.irsst.qc.ca/mixie/?en (Québec version) http://www.inrs.fr/publications/outils/mixie/calculateur.html (French version)	web	free	active	Québec version : Institut de Recherche Robert Sauvé en Santé au Travail, University of Montréal French version : Institut de Recherche Robert Sauvé en Santé au Travail, French Research and Safety Institute for the [illegible]	French									
**NanoRiskCat / Nano-Database**	NanoRiskCat / Nano-Database	Consumer, worker	Oral, dermal, inhalation	activities with nanomaterials	Nanomaterials, consumer products, products for professional end-users	0	generic in nature and can be used on all kinds of nanomaterials and applications	does not it take into account the specific content of nanomaterial in the product.		http://www.nanowerk.com/spotlight/spotid=24072.php					english									
**NanoSafer**	NanoSafer	Worker	inhalation	activities with nanomaterials	Engineered nano materials		Is flexible in that you can combine different materials (with their characteristics and hazards) along with different processes to determine the level of protection needed.	Software is in English, but support documents are in Danish		http://www.nanosafer.org/	Web-based		active	NanoSafer: National Research Center for the Working Environment, Denmark and the Danish Nanosafety Center	english								2020	
**OH-MOD**	OH-MOD	Different Targets predominately industrial hygiene	inhalation	activities with chemicals	The oxygen content trends in environments where inert gas releases may lead to asphyxiation risks	Tier 1 to 3 (depends on parametrisation)	Built from a standard modeling approach (2-zone inhalation exposure model published in AIHA's modeling guide); probabilistic model; assesses long-term and acute exposures	Estimating some inputs may be difficult for novice users.		https://www.aiha.org/public-resources/consumer-resources/topics-of-interest/ih-apps-tools	Excel	free	active	AIHA's Exposure Assessment Strategies Committee (EASC)	Multilingual interface	quantitative values	Differential equations based on physical-chemical laws	Quantitative values	yes	yes	Estimates the oxygen concentration percent by volume and as mmHg partial pressure over time in the two zones	1	2021	JK

**Precautionary Matrix for Synthetic Nanomaterials**	Swiss Precautionary Matrix for Synthetic Nanomaterials	Different Targets	inhalation, dermal	activities with nanomaterials	Engineered nano materials		Inputting data is relatively easy and are well explained. The outputs are very descriptive and provide good information.	Data will expire in 20 minutes after last page view, so must save work periodically.		https://www.bag.admin.ch/bag/en/home/gesund-leben/umwelt-und-gesundheit/chemikalien/nanotechnologie/sicherer-umgang-mit-nanomaterialien/vorsorgeraster-nanomaterialien-webanwendung.html	Web-based and downloadable application	Downloadable version: https://www.bag.admin.ch/bag/en/home/gesund-leben/umwelt-und-gesundheit/chemikalien/nanotechnologie/sicherer-umgang-mit-nanomaterialien/vorsorgeraster-nanomaterialien-downloadversion.html		Precautionary Matrix: Federal Office of Public Health, Switzerland	english									
**PROWESE**	PRObabilistic Web-based model for Estimating Systemic Exposure	Worker	Systemic Exposure		several	distribution for single exposure, goal was to also allow cumulative exposure assessment (not implemeted)		Model was set up as a webbased tool (running on a Matlab server). But currently does not appear to be online anymore		https://www.hse.gov.uk/research/rrpdf/rr763.pdf				HSE										
**RISKOFDERM**	RISKOFDERM 2.1	Worker	dermal	activities with dermal exposure	chemicals	higher tier	only model which covers body exposure, industrial and professional uses covered	Performance of protective clothing and gloves has to be introduced externally to the model to produce an estimate of actual dermal exposure (ADE) which can be used to compare with an external DNEL. Restrictions due to original data set and fumes are not covered. Far-field factors are not included. [illegible]	Former versions were evaluated. Warnings were added to indicate that use rates lower than 1 L/min should not be used in the model for Filing, mixing and loading for liquids.	https://echa.europa.eu/documents/10162/19680902/calculator_riskofderm_enl.xls/9e0c3fa8-4764-4a18-95f9-8fbccf3acf2a	Excel spreadsheet version	free	inactive	TNO/HSL	englisch	qualitative expressions, quantitative values	regression analysis	several percentiles	yes	yes	Initial development	2.2.1	2014	JM

**SEIRICH**	Tool for Risk assessment and information in occupationnal settings (in French Système d'Evaluation et d'Information sur les Risques Chimiques en milieu professionnel)	worker, environment	inalation, dermal, fire explosion		chemicals		assesses chemical risk (hazard ADN exposure), simple of use, Inputs are easy to select and determine; simple structure.	doesnt evaluate situation with asbestos the storage task is not included in the seirich task catalogue		https://www.seirich.fr/seirich-web/comprendrelesrisqueschimiques.xhtml	windows	free	active(V 3.0.0)	developed and diffused by the French Research and Safety Institute for the Prevention of Occupational Accidents and Diseases (INRS), together with national partners.	Frensh/ englisch									
**SprayExpo**	SprayExpo	Worker	inhalation, dermal	spraying activities with chemicals	Biocides	Detailed, realistic assessment (higher Tier)	It is assumed that the sprayed product is composed of a non volatile substance dissolved in a solvent with known volatility. The model is based on a simulation of the motion of released droplets taking into account gravitational settling, turbulent mixing with the surrounding air, and [illegible]	With regard to dermal exposure, the model can only take the sedimentation flow of the airborne droplets into account, but not accidentally occurring splashes. Therefore, in the majority of cases, the dermal exposure at the workplace is underestimated by SprayExpo. In the case of room spraying, [illegible]		https://www.baua.de/media/BAuAInternet/download/SprayExpo2.3.xls	Windows® XP with Service Pack 2 or later– Microsoft Excel® 2007 or later	free		Federal Institute for Occupational Safety and Health (BAuA), Germany	englisch, german	quantitative values, qualitative expressions	Differential equations based on physical-chemical laws	distinct values, distinct time resolved values	yes	yes		2.3	2016	JM
**Stoffenmanager Nano - module 1.0**	Stoffenmanager Nano - module 1.0	Worker	inhalation	activities with nanomaterials	Engineered nano materials; Includes agglomerates and aggregates The size of the primary particle is smaller than 100 nm and / or the specific surface area of a nanopowder is larger than 60 m2/g;		Inputs are easy to select and determine. Can determine the impact on the exposure and risk class of changing various control measures.	ENP cannot be water soluble		https://nano.stoffenmanager.com/	Web-based			Stoffenmanager Nano: Cosanta B.V.	englisch, nederlands									

**STOFFENMANAGER®**	STOFFENMANAGER® 8	Worker	inhalation, dermal	activities with chemicals	The model differentiates between different exposure processes: vapor, mist, and dust. Fumes, fibers, and gases are not considered by the model	Tier 1.5	In general STOFFENMANAGER© offers – for a Tier 1.5 tool – a high level of detail and thus, allows for a good description of the exposure situation. STOFFENMANAGER© is the only tool within this project which describes background factors and far-field factors in a transparent way and covers all relevant [illegible]	Exposures to fibres, gases or substances released into the air as an effect of welding or soldering are outside the scope of the tool. Assessments for abrasion and impact of solid objects are only possible for stone and wood. Exposure to respirable dusts is only implemented [illegible]	several validation studies, e.g. ETEAM + peer-review studies to explore the validity (reliability) of the exposure estimates	www.stoffenmanager.com		free, needs registration (+ paid versions available)	active, continuous updates available	Cosanta BV	available in several languages: dansk, deutsch, english, español, français, italiano, nederlands, polski, português, suomi, svenska	qualitative expressions, quantitative values, value bands	modifying factors and subsequent calibration	distinct values	yes	yes	model	8.3	2020	JM
**TEAS**	Task Exposure Assessment Simulator	Different Targets predominately industrial hygiene. Applicable also for consumer exposure assessment.	inhalation	activities with chemicals	vapour, particles	Tier 1 to 3 (depends on parametrisation)	TEAS is a program – Windows 7, 8, and 10 only – for predicting near and far field exposures and exposure profiles, using the standard and new Well-Mixed Room (WMR) models and modern probabilistic modeling methods. TEAS includes popular algorithms for predicting generation rates, near field [illegible]		Validated (e.g. Jayjock et al. 2011; Abattan et al. 2020)	https://www.easinc.co/teas-software/	Application	Commercial	active	Exposure assessment solutions, Inc.	English	quantitative values, distributions	Differential equations based on physical-chemical laws	Quantitative values	yes	yes	Estimate current exposures: compare predictions to TWA OELs, STELs, Ceiling Limits, IDLH limits, and LELs. Predict future exposures: prospective exposure assessment (evaluate proposed changes to the process and production level). [illegible]	1	2019	JK

**TnsG**	Technical notes for guidance - human exposure to biocidal products - guidance on exposure estimation (TNsG 2002, TNsG 2007)	Worker	inhalation, dermal	activities with biocides	biocides	higher tier	based on measurement data	confined to specific exposure situations		https://echa.europa.eu/documents/10162/16960215/bpd_guid_tnsg+human+exposure+2002_en.pdf/af2020f7-6cd2-471a-8cf2-efd1a0500fa8 https://www.echa.europa.eu/documents/10162/16960215/bpd_guid_tnsg-human-exposure-2007_en.pdf/3d59996f-b548-4bf0-876b-e04b5db04617	document	Free		ECHA?	English	application duration, amount of active substance handled, number of cycles	read-across	several percentiles	no	yes		version 2002, version 2007	2007	JM
**TREXMO**	TRanslation of EXposure MOdels	Worker	inhalation	activities with chemicals	chemicals	Tier 1 and 2	Multi-models approach that allows to run simultaneously exposure assessment in different models for a given, same, exposure situation. Easy comparison of different exposure estimates.	Does not provide recommendation on which exposure estimate should be used for the risk assessment	the different tools that are included were evaluated frequently	TREXMO 2.0: http://trexmo.chuv.ch	Web-based tool	Free		Unisanté (Lausanne, CH), SECO (Bern, CH)	English	value bands, qualitative expressions, quantitative values	read-across	several percentiles, value ranges, distinct values, advanced statistics	yes	no	ART v.1.5, Stoffenmanager® Version 4.0 (Schinkel et al. 2010), ECETOC TRA v.3, MEASE v.1.02.01, EMKG-EXPO-TOOL und EASE v.2.0. Version 3 expected to include more user-friendly and modern interface and another exposure model, TREXMO+.	2.0	2016	JM
**TREXMO+**	TREXMO Plus (TREXMO+)	Worker	inhalation	activities with chemicals	chemicals	Tier 2+	Its concepts ensures better performance compared to the existing REACH models	Does not calculate higher percentiles (e.g. 90th)		https://www.nature.com/articles/s41370-020-0203-9	R software (intended to be part of TREXMO 3)	Unknown		Unisanté (Lausanne, CH), SECO (Bern, CH)	English, French, German, Italian	value bands, qualitative expressions, quantitative values	machine learning		yes	no	machine learning as a method to continuously evaluate the performance of different exposure models.			JM

short name	name of the model / tool	exposure target	route of exposure	sources of exposure	product class / chemicals / substances	tier / complexity	strengths	limitations	evaluation status	source / reference / download	platform	availability	level of maintenance	owner / developer	language	model input	model structure	model output	tool	model	remarks on model / tool	version available	last update	edited by
**ADL AMEM**	ADL Polymer Migration Estimation Model (AMEM)	Different Targets	inhalation, dermal	articles			realistic migration rates (note very old - MS DOS)		http://www.epa.gov/oppt/exposure/pubs/amemdl.htm		free			englisch							estimation of the fraction of the additive originally in the polymer sheet that			
**AISE REACT**	AISE REACT Consumer Tool	Consumer	inhalation, dermal, oral	Household Products		Based on industry H&P data. Some scenarios are known to be conservative		Europe		https://www.aise.eu/our-activities/product-safety-and-innovation/reach/consumer-safety-exposure-assessment.aspx	Excel spreadsheet	free			english	Quantitative values	linear equations	Quantitative values	yes	yes		1	2010	NvG
**APEX**	Air Pollutants Exposure Model	Humans	inhalation	outdoor, indoor, in-vehicle	air pollutants	Tier 3	Realistic and flexible. Enables modelling of vulnerable populations within the bulk population	Does not consider interactions between modelled pollutants; Modelling domain size roughly a large city's metropolitan area; default input files parameterized for the US	Johnson et al., 2018: https://doi.org/10.1080/23311843.2018.1453022	https://www.epa.gov/fera/human-exposure-modeling-air-pollutants-exposure-model	Windows-based executable	free	active	United States Environmental Protection Agency (EPA)	English	quantitative values, distributions		quantitative values, distributions	yes	yes	Some defaults based on US data, but model fully customizable to a non-US-based location. Used for setting air quality standards for air pollutants	5,2		NvG
**BROWSE model**	see sheet "Worker"	Worker, general population	inhalation, dermal, oral	Plant protection products, pesticides																				
**Calendex**	Calendex	General population	inhalation, dermal, oral	food, drinking water, environmental exposure	pesticides and other chemicals	Tier 3		US	Benchmarking with other models available.	https://www.epa.gov/pesticide-science-and-assessing-pesticide-risks/deem-fcidcalendex-software-installer		free		US-EPA/ Exponent	english							10		NvG
**CARES**	CARES	Consumer	inhalation, dermal, oral		pesticides, single/aggregate/cumulative	Tier 1, 2 and 3		US	Benchmarking with other models available.	https://www.epa.gov/pesticide-science-and-assessing-pesticide-risks/models-pesticide-risk-assessment		free		US-EPA	english						older Version of CARES NG			NvG

**CARES NG**	Creme Cumulative and Aggregate Risk Evaluation System Next Generation (CARES NG)	Consumer	oral		Pesticides					https://www.cremeglobal.com/products/cares-ng		Commercial software	active	Creme Global	english	quantitative values, distributions		quantitative vaues, distributions			based on CARES (free software)			NvG
**CEM**	Consumer Exposure Model	Consumer	inhalation, dermal, oral	products/materials in the indoor environment						https://www.epa.gov/tsca-screening-tools/cem-consumer-exposure-model-download-and-install-instructions	Microsoft Access and Visual Basic for Applications (VBA)	free		US-EPA	english							2,1		NvG
**Chesar**		Consumer	inhalation, dermal, oral							https://chesar.echa.europa.eu/		free		European Chemicals Agency (ECHA)					yes	no	based on Ecetoc TRA	3,6	2021	NvG
**ConsExpo**	ConsExpo / ConsExpo web	Consumer	inhalation, dermal, oral	Consumer products, e.g. paint, cleaning agents, personal care product; vapour and particulates		Tier 2	Data and scenarios can be saved. Fore specific scenarios, necessary parameters can be extracted from ConsExpo fact sheets, which have recently been updated by a consortium.	Comparatively many parameters. For some parameters, default values given	Benchmarking with other models available. Some modules tested against experimental data, e.g. the spray module (Delmaar & Bremmer, 2009, RIVM rapport 320104005) and the PC&P module used by PACEM [illegible]	https://www.rivm.nl/en/Topics/C/ConsExpo https://www.rivm.nl/en/Documents_and_publications/Scientific/Reports/2016/december/ConsExpo_Web_Consumer_exposure_models_Model_documentation	web-based	free	Active – several versions, continuous updates available from RIVM (NL) webpage	Dutch National Institute for Public Health and the Environment (RIVM)	english	Quantitative values	Differential equations based on physical-chemical laws	Quantitative values	yes	yes		4,1	2016	NvG
**Creme Care & Cosmetics**	Creme Care & Cosmetics	Consumer	inhalation, dermal, oral	Personal care and cosmetic products	cosmetic ingredients	Tier 2 & 3				https://www.cremeglobal.com/products/creme-care-cosmetics		Commerical software	active	Creme Global	english	quantitative values, distributions	Monte Carlo simulations	quantitative values, distributions			Page not found			NvG
**Creme Food Safety**	Creme Food Safety	Consumer	oral	Food	Food constituents and ingredients	Tier 3	Probabilistic model based on food consumption surveys. Regulator Acceptance. Scenario Analysis with detailed statistical analysis			https://www.cremeglobal.com/products/creme-food-safety		Commercial software	active	Creme Global	english	quantitative values, distributions	Monte Carlo simulations	quantitative values, distributions	yes	yes				NvG

**Creme Nutrition**	Creme Nutrition	Consumer	oral	Food	nutrients	Tier 3	Probabilistic model based on European food consumption data. Full distribution of intake broken down by foods or groups of foods.			https://www.cremeglobal.com/creme-nutrition/		Commercial software		Creme Global	english	quantitative values, distributions	Monte Carlo simulations	quantitative values, distributions	yes	yes				NvG
**Creme RIFM**	Creme RIFM	Consumer	inhalation, dermal, oral	personal care and cosmetic products	Fragrance compounds	Tier 2 & 3	Probabilistic, individual-based model, based on population use data			https://www.cremeglobal.com/products/creme-rifm		Commerical software on cloud-based platform.	active	Creme Global	english	quantitative values, distributions	Monte Carlo simulations	quantitative values, distributions	yes	yes				NvG
**DEEM-FCID**	Dietary Exposure Evaluation Model - Food Commodity Intake Database	General population	oral	Food	pesticides	Tier 1 and 2		US		https://www.epa.gov/pesticide-science-and-assessing-pesticide-risks/deem-fcidcalendex-software-installer		free			english							4,02		NvG
**DIAMOND**	DIAMOND	Consumer	oral	Food	pesticides, nutrients, contaminants, food additives and food ingredients	unknown		Australia and New Zealand		https://www.foodstandards.gov.au/science/exposure/Pages/fsanzdietaryexposure4439.aspx		restricted use	old model, under re-evaluation	Food Standards Australia New Zealand (FSANZ)	english									NvG
**DustEx**	DustEx tool	Consumer	inhalation, dermal, oral	Consumer products via the indoor environment (dust, air)	Semi-volatile organic compounds (SVOCs)	Tier 2				https://www.dustex.nl/	web-based	free	active	RIVM	english				yes	yes		1	2017	NvG
**dynamiCROP**	dynamiCROP: dynamic assessment model for human health impacts due to CRop uptake of Organic Pollutants	Consumer	ingestion	Food	organic contaminants	screening level, quantitative, dynamic analysis of chemical residues in food crops; includes Monte Carlo uncertainty analysis	accounts for dynamics of chemicals in plant-environment system until crop harvest; considers parameterizations for various food and feed crops		Crop residue estimates evaluated for different food crops against measured residue data	http://dynamicrop.org	Excel spreadsheet, coupled to Matlab and Python for ODE solvers	free	active	Technical University of Denmark (DTU)	english	Quantitative values	Differential equations based on physical-chemical laws, structured in matrices (e.g. for rate constants) and vectors (e.g. initial mass conditions)	quantitative values	yes	yes	Included as parameterized version for 6 crop archetypes in the UNEP-SETAC global scientific consensus model USEtox; version for LCA available	3,12	2021	
**EASY-TRA**	see sheet "Worker"	Consumer, worker, environment	inhalation, dermal, oral		several																			

**Ecetoc TRA**	Ecetoc TRA	Consumer (also modules for workers and environment available)	inhalation, dermal, oral	consumer products		Tier 1, 1.5, 2	Widely accepted in European regulation, large variety of product applications covered	Gases and fibres out of scope, not directly applicable to molten solids used at elevated temperature. Inhalation exposure to liquid aerosols not covered. For spray processes using liquids, only vapour exposure estimated.	several validation studies, e.g. ETEAM + peer-review studies to explore the validity (reliability) of the exposure estimates	https://www.ecetoc.org/tools/targeted-risk-assessment-tra/	Excel spreadsheet	free		CEFIC, Ecetoc		Quantitative values	linear equations	Quantitative values				3,1	2014	NvG
**E-FAST**	E-FAST (Exposure and Fate Assessment Tool)	Consumer	inhalation, dermal, oral	Vapor and particulates			built-in examples. Considers essentially all major routes of exposure Model can incorporate different environments indoor (residence, office, school, or automobile) and outdoor Model can estimate parameters	Many parameters needed, but most can be estimated within the model or using EpiSuite. .	consumer exposure portions of E-FAST have been peer reviewed by experts outside EPA	https://www.epa.gov/tsca-screening-tools/e-fast-exposure-and-fate-assessment-screening-tool-version-2014	Access	free		US-EPA - Office of Pollution Prevention and Toxics	english				yes	yes		2	2014	NvG
**EPA ExpoBox**	US EPA Exposure tool box	Different Targets, Overview platform	inhalation, dermal, oral		chemicals					https://www.epa.gov/expobox https://cfpub.epa.gov/ncea/risk/expobox/efhToolSearch.cfm			active	United States Environmental Protection Agency (EPA)	english						Continuously updated			
**ESIG EGRET**	ESIG EGRET Consumer Exposure Tool (GES/CSA tool)	Consumer	inhalation, dermal	Household Products, solvents		conservative			http://www.nature.com/jes/journal/v24/n1/full/jes2012128a.html	https://www.esig.org/reach-ges/consumers/		free		European Solvents Industry Group (ESIG)	english							2,1	2017	NvG
**EUSES**	European Union System Evaluation Substances	General population, environment	inhalation, oral, environmental compartments (water, wastewater)		chemicals	screening level	multi-compartment calculations, consideration of all uses of a substance within the assessment (regional / continental environmental concentrations)	Currently not applicable for anorganic substances, nanomaterials, substances that are ionisable under environmental conditions. Does not take into account sector-specific RRMs.		https://echa.europa.eu/de/support/dossier-submission-tools/download-euses		free		RIVM and European Commision	englisch							2.1.2		NvG

**ExpoCast**	ExpoCast: Exposure Science for Prioritization and Toxicity Testing	General population	inhalation, dermal, oral		Environmental pollutants		Tool that combines Near-field (SHEDS) and Far-field (RAIDAR) models			https://cfpub.epa.gov/si/si_public_record_Report.cfm?dirEntryId=211811		free	active	United States Environmental Protection Agency (EPA)										NvG
**FACET**	FACET	General population	oral	Aggregate and single product flavours, additives and food contact materials		Tier 3		based on EU registrations and legislation		https://ec.europa.eu/jrc/en/scientific-tool/flavourings-additives-and-food-contact-materials-exposure-tool		free		JRC	english	quantitative values, distributions	Monte Carlo simulations	quantitative values, distributions				3.0.2		NvG
**FAIM**	Food Enzyme Intake Model	General population	oral	Aggregate and single product		Tier 1	comprises process-specific calculators for food processing	based on EU registrations and legislation		https://zenodo.org/record/154725#.YGXgGGixXOQ	Excel spreadsheet	free		EFSA	english				yes	yes		1,1	2013	NvG
**FHX model**	FHX (Far-field Human eXposure) model	General population	inhalation, dermal, oral		organic chemicals	Screening				https://arnotresearch.com/fhx/		Free, registration needed	complete	ARC Arnot Research	englisch									NvG
**ICECRM**	The Indoor Chemical Exposure Classification/Ranking Model	General population	topical, inhalation, oral (non dietary exposure)	indoor multimedia			realistic concentrations			https://arnotresearch.com/ICECRM/	The ICECRM model is implemented (coded) in Visual Basic for Applications (VBA) and the Graphical User Interface is designed in Excel™.	free	active	ARC	english									
**INTERA**	INTERA	Consumer	inhalation, dermal, oral	articles		realistic, including uncertainty analysis				http://www.intera-home.eu/		Free, registration needed		Consortium	english									NvG
**iRISK**	iRISK	Consumer	oral	Food	chemicals and pathogens	Tier 1 and 2				https://irisk.foodrisk.org/		free		Food and Drugs Agency (FDA)	english	quantitative values, distributions	Monte Carlo simulations, also 2D	quantitative values, distributions	yes			4,2		NvG
**MCCEM**	MCCEM	Consumer	inhalation	released from articles	environmetal /indoor air					http://www.epa.gov/opptintr/exposure/pubs/mccem.htm		free			english						calculated as single day doses, chronic average daily doses, or lifetime average daily doses			

**MCRA tool**	Monte Carlo Risk Assessment tool	General population	oral	Food		Tier 3	Probabilistic model based on European food consumption data. Full distribution of intake broken down by foods or groups of foods.			https://mcra.rivm.nl		Free, registration needed	active	RIVM/Rikilt	english	quantitative values, distributions	Monte Carlo simulations, also 2D	quantitative values, distributions	yes	yes		9		NvG
**Merlin-Expo tool**	**M**odelling **E**xposure to chemicals for **R**isk assessment: a comprehensive **L**ibrary of multimedia and PBPK models for **I**ntegration, u**N**certainty and sensitivity analysis	General population	inhalation, dermal, oral	Environmental sources	Environmental pollutants		Allows lifetime risk assessments (rather than just simple daily intakes) for different human populations including exposure through multiple pathways			https://merlin-expo.eu/		free	active	EU	english									NvG
**MOEEBIUS**	MOEEBIUS	Energy optimization and air quality	inhalation	activities with chemicals	vapour, particles	Tier 1 to 2 (depends on parametrisation)			Validated (single compartment model)	https://www.moeebius.eu/	Application	free	active	AIHA's Exposure Assessment Strategies Committee (EASC)	English	quantitative values, distributions	Differential equations based on physical-chemical laws	Quantitative values	No	yes			2017	JK
**NanoRiskCat / Nano-Database**	see sheet "Worker"	Consumer, worker	Oral, dermal, inhalation		Nano specific		generic in nature and can be used on all kinds of nanomaterials and applications																	
**NanoRiskCat / Nano-Database**	see sheet "Worker"	Consumer, worker	Oral, dermal, inhalation		Nano specific	generic in nature	can be used on all kinds of nano materials and applications																	
**PACEM**	Probabilistic Aggregate Consumer Exposure Model	Consumer	inhalation, dermal, oral	Cosmetics and personal care products; household cleaning products		Tier 2 & 3	Probabilistic, individual-based model, based on population use data			https://zenodo.org/deposit/1475191.	R-Shiny	free R-Shiny beta version available, free web-version under development		Gosens et al. (2014), Delmaar et al. (2014), Dudzina et al. (2015), Karrer et al. (2019)	english	quantitative values, distributions	Monte Carlo simulations	quantitative values, distributions	no	yes		beta	2019	NvG

**Pangea**	Pangea	General population	inhalation, oral	industrial releases, agricultural applications	organic chemicals	screening level, quantitative, geospatialized analysis	global applicability; can be adapted to any spatial region and resolution; only model for chemical pollution that provides a flexible spatial grid resolution with global coverage	maximum resolution is limited to scales for which 2nd order processes can be neglected	Spatial model output evaluated in case studies against measured environmental concentrations; Underlying processes based on USEtox, and with that evaluated via several in-depth model comparisons between 2002-2008	http://www.pangea-model.org	Matlab (math engine), ArcGIS (geospatialization engine), Python (processing engine)	only accessible by model owners	active	Technical University of Denmark (DTU)	english	Quantitative values	Differential equations based on physical-chemical laws, structured in matrices (e.g. for rate constants) and vectors (e.g. emission sources)	Quantitative values	yes	yes	Enviornmental and exposure processes entirely based on UNEP-SETAC global consensus model USEtox	2.0	10/19	
**PiF Model**	Product Intake Fraction (PiF) Model	Consumer	inhalation, dermal, oral	direct indoor/outdoor emissions, industrial releases	organic chemicals, chemicals in consumer products	quantitative; builds on dynamic solutions of direct consumer exposure	global applicability; parameterized for situations where specific household or other exposure settings are unknown; steady-state, but underlying product models are dynamic	Steady-state solution for overall mass balance	Framework based on several modules for products and indoor exposure that have all been evaluated against measurement data	http://doi.org/10.1016/j.envint.2016.06.010	Excel spreadsheet (underlying product models implemented in Excel or Matlab)	free	active (as implemented in USEtox 3.x)	Technical University of Denmark (DTU)	english	Quantitative values	Differential equations based on physical-chemical laws, structured in matrices (e.g. for rate constants) and vectors (e.g. sources)	Quantitative values	yes	yes	Recommended by UNEP-SETAC Life Cycle Initiative for use in LCA and comparative risk screening	1.0	10/17	
**PRIMo**	Pesticide Residue Intake Model	General population	oral		pesticides, single product only	Tier 1		EU		https://www.efsa.europa.eu/en/applications/pesticides/tools	Excel spreadsheet with Macros	free		EFSA	english	Quantitative values		Quantitative values	yes	yes		3,1	2018	NvG
**RAIDAR**	Risk Assessment IDentification And Ranking (RAIDAR) model	General population, environment	inhalation, oral		Organic chemicals	Prioritization and screening-level assessments	High-throughput exposure and risk estimation	Regional scale, evaluative enviroment	Arnot, J. A. et al. ES&T, 2006, 40, (7), 2316-2323; Arnot et al. EHP, 2012, 120, (11), 1565-1570.	https://arnotresearch.com/RAIDAR/	Excel/VBA	free, registration needed	active	ARC Arnot Research and Consulting Inc.	english				yes	yes		2,02	2014	NvG

**RAIDAR-ICE**	Risk Assessment IDentification and Ranking – Indoor and Consumer Exposure (RAIDAR-ICE) Model	General population	inhalation, topical/ dermal, oral	near field direct and indirect exposure	neutral organic chemicals	realistic concentrations<br><br>Prioritization and screening-level assessments	High-throughput and screening-level exposure and risk estimation	Currently applicable for discrete neutral organic chemicals	Li, L.; Westgate, J. N.; Hughes, L.; Zhang, X.; Givehchi, B.; Toose, L.; Armitage, J. M.; Wania, F.; Egeghy, P.; Arnot, J. A. Environ. Sci. Technol. 2018, 52, (24), 14235-14244.	https://arnotresearch.com/RAIDAR-ICE/	online<br><br>Excel/VBA	free, registration needed	active	ARC Arnot Research and Consulting Inc.	english				yes		RAIDAR-ICE is part of the US EPA's ExpoCast System for the Empirical Evaluation of Models (SEEMS) human exposure framework			NvG
**RIFM 2-Box**	RIFM 2-Box Indoor Air Dispersion Model	Consumer	inhalation	Cosmetics and personal care products		Tier 1 or 2				https://www.rifm.org/rifm-science-assessment-tools.php#gsc.tab=0		restricted use, RIFM database subscription needed												NvG
**RIVM Emission tool**	RIVM Emission tool	Consumer	inhalation	articles		realistic air concentrations				www.consexpo.com		free			english									
**SHEDS-HT**	SHEDS-high throughput	General population	inhalation, dermal, oral			screening level				https://www.epa.gov/chemical-research/stochastic-human-exposure-and-dose-simulation-sheds-estimate-human-exposure	SAS 9.1 or higher	free, registration needed	active	United States Environmental Protection Agency (EPA)	english	quantitative values, distributions		quantitative values, distributions	yes	yes		beta		NvG
**SHEDS-Multimedia**	Stochastic Human Exposure and Dose Simulation (SHEDS) model	General population	inhalation, dermal, oral	Food, residential exposures		screening level, Tier 2 & 3				https://www.epa.gov/chemical-research/stochastic-human-exposure-and-dose-simulation-sheds-estimate-human-exposure	SAS 9.1 or higher	free, registration needed	active	United States Environmental Protection Agency (EPA)	english	quantitative values, distributions		quantitative values, distributions	yes	yes		v3		NvG
**SprayExpo**	see sheet "Worker"	Consumer		Biocides	Biocidal substances																			

**SWIMODEL**	SWIMODEL	General population	oral, dermal, inhalation, buccal/sublingual, nasal/orbital, aural	water indoor swimming pools and spas	biocides, pesticides					https://www.epa.gov/pesticide-science-and-assessing-pesticide-risks/swimmer-exposure-assessment-model-swimodel	excel spreadsheet	free			english									NvG
**USEtox**	The UNEP-SETAC scientific consensus model for characterizing human and ecotoxicological impacts of chemical emissions in life cycle assessment	General population, consumers, specific population groups (e.g. children)	inhalation, dermal, oral	global applicability; parameterized for situations where emission locations are unknown, and parameterized (sub-)continental regions	organic chemicals and metal ions, industrial releases, agricultural emissions	screening level	steady-state and dynamic version available, far-field and near-field exposure modules	global parameterization model with limited applicability to local situations	Evaluated via several in-depth model comparisons between 2002 and 2008	https://usetox.org	Excel spreadsheet and Matlab version (for internal use)	free	active	USEtox International Centre hosted at the Technical University of Denmark (DTU)	engliseh	Reference model in life cycle impact assessment and environmental footprinting	Differential equations based on physical-chemical laws, structured in matrices (e.g. for rate constants)	impact characterization factors including fate, exposure, and effect factors	yes	yes	Endorsed by UNEP-SETAC Life Cycle Initiative for use in LCA and comparative risk screening; recommended by EU (ILCD) and US-EPA (TRACI)	v2.12, v3beta	03/22	

short name	name of the model / tool	exposure target	route of exposure	sources of exposure	product class / chemicals / substances	tier / complexity	strengths	limitations	evaluation status	source / reference / download	platform	availability	level of maintenance	owner/ developer	language	model input	model structure	model output	tool	model	remarks on model / tool	version available	last update	edited by
**BEEHAVE**	BEEHAVE	Bees	Feed	pollen, nectar	pesticides					https://beehave-model.net/		free		Research team	english									NvG
**ECETOC TRA**	see sheet "Worker"	Environment (also modules for worker and consumers available)	inhalation, dermal, oral		liquid and solid chemicals																			
**EUSES**	see sheet "general population"																							
**EXAMS**	Exposure Analysis Modeling System (EXAMS)	Aquatic species	aquatic ecosystem models and rapidly evaluating the fate, transport, and exposure concentrations, water		Environmental pollutants		EXAMS contains an integrated Database Management System (DBMS) specifically designed for storage and management of project databases required by the software. User interaction is provided by a full featured Command Line Interface (CLI), context-sensitive help menus, an on-line data [illegible]			https://www.epa.gov/exposure-assessment-models/exams-version-index			active	United States Environmental Protection Agency (EPA)	english									
**GREAT-ER 4.0**	Geo-referenced Regional environmental Exposure Assessment Tool for European Rivers (GREAT-ER) 4.0	Aquatic species	river, water		“down-the-drain” chemicals					http://cefic-lri.org/toolbox/great-er/	GREAT-ER 4.0 running under MS Windows as Add-In of ArcGIS Desktop 10 (ESRI®)		complete	Kehrein et al., 2015	english	quantitative values	system of differential equations	quantitative values	yes			4		NvG
**iSTREEM model**	iSTREEM model	Aquatic species	effluent, receiving waters		“down-the-drain” chemicals					https://www.cleaninginstitute.org/industry-priorities/science/istreem			complete	The American Cleaning Institute (ACI)	english									NvG

**OECD Pov and LRTP Screening Tool**	OECD overall persistence (Pov) and long-range transport potential (LRTP) Screening Tool	Environment	multimedia chemical fate models		organic chemicals	screening	The Tool software allows a simple sensitivity and uncertainty analysis of results for the single chemical; Monte Carlo Analysis for Single Chemical			http://www.oecd.org/env/ehs/risk-assessment/oecdpovandlrtpscreeningtool.htm	The Tool is a Microsoft Excel file with included Visual Basic code. The program code and input and output data are integrated with the spreadsheet functions of Excel.		complete		english									
**Pangea**	Pangea	aquatic and terrestrial species	multimedia chemical fate model, and dissolved chmical fraction in exposure environments	industrial releases, agricultural applications	organic chemicals	screening level, quantitative, geospatialized analysis	global applicability; can be adapted to any spatial region and resolution; only model for chemical pollution that provides a flexible spatial grid resolution with global coverage	maximum resolution is limited to scales for which 2nd order processes can be neglected	Spatial model output evaluated in case studies against measured environmental concentrations; Underlying processes based on USEtox, and with that evaluated via several in-depth model comparisons between 2002-2008	http://www.pangea-model.org	Matlab (math engine), ArcGIS (geospatialization engine), Python (processing engine)	only accessible by model owners	active	Technical University of Denmark (DTU)	english	Quantitative values	Differential equations based on physical-chemical laws, structured in matrices (e.g. for rate constants) and vectors (e.g. emission sources)	Quantitative values	yes	yes	Enviornmental and exposure processes entirely based on UNEP-SETAC global consensus model USEtox	2.0	10/19	
**RAIDAR**	see "General population (human)"														english									
**TERRACE**	TERRACE, Terrestrial Runoff Modelling for Risk Assessment of Chemical Exposure	Environment	waters entering river systems		Environmental pollutants					http://cefic-lri.org/toolbox/terrace/			Complete - The TERRACE database is no longer compatible with current operating systems.	by CEFIC LRI in collaboration with Cranfield University and University of Durham	english									
**USEtox**	The UNEP-SETAC scientific consensus model for characterizing human and ecotoxicological impacts of chemical emissions in life cycle assessment	aquatic and terrestrial species	multimedia chemical fate model, and dissolved chmical fraction in exposure environments	global applicability; parameterized for situations where emission locations are unknown, and parameterized (sub-)continental regions	organic chemicals and metal ions, industrial releases, agricultural emissions	screening level	steady-state and dynamic version available	global parameterization model with limited applicability to local situations	Evaluated via several in-depth model comparisons between 2002 and 2008	https://usetox.org	Excel spreadsheet and Matlab version (for internal use)	free	active	USEtox International Centre hosted at the Technical University of Denmark (DTU)	english	Reference model in life cycle impact assessment and environmental footprinting	Differential equations based on physical-chemical laws, structured in matrices (e.g. for rate constants)	impact characterization factors including fate, exposure, and effect factors	yes	yes	Endorsed by UNEP-SETAC Life Cycle Initiative for use in LCA and comparative risk screening; recommended by EU (ILCD) and US-EPA (TRACI)	v2.12, v3beta	03/22	

short name	name of the model / tool	exposure target	route of exposure	sources of exposure	product class / chemicals / substances	tier / complexity	strengths	limitations	evaluation status	source / reference / download	platform	availability	level of maintenance	owner/ developer	language	model input	model structure	model output	tool	model	remarks on model / tool	version available	last update	edited by
**GastroPlus**	Gastroplus incl. PBPKPlus/ADMET predictor/ect.	Consumer	inhalation, dermal, oral		drugs					https://www.simulations-plus.com/software/gastroplus/		commercial	active	Simulations Plus	english				yes	yes		9.8.1	2021	NvG
**HBM SIMULATOR**	Human Biomonitoring (HBM) SIMULATOR	Consumer	inhalation, dermal, oral		4 different metals (As, Cd, Mn, Ni) and 15 organic compounds (incl. 9 parabens, triclosan, triclocaban, chlorophenone-1, -3, -8, and Bisphenol A)					http://cefic-lri.org/toolbox/hbm-simulator/	Runs on MATLAB Component Runtime 7.14 needs to be installed.	free	active	Cefic LRI / VITO / the Health & Safety Laboratory and the Summit Toxicology	english									NvG
**HTTK**	High-Throughput Toxicokinetics	Consumer	oral		pharmaceuticals					https://CRAN.R-project.org/package=httk	R software	free		Pearce RG, Setzer RW, Strope CL, Wambaugh JF, Sipes NS. httk: R Package for High-Throughput Toxicokinetics. *J Stat Softw* . 2017;79(4): 1-26. doi:10.18637/jss.v079.i04	english							1.7		NvG
**IH SKINPERM**	IH SKINPERM	Worker, Consumer	dermal		Liquid, vapor, and solids	Includes a built-in database that allows user to select from a list of chemicals. The model is easy to use, easy to read and comprehend reports, includes the ability to add chemicals and create a User's Database		Pure substances or aqueous mixtures only, unless the water solubility of the substance in the non-aqueous mixture is known. Molecular weight between 18 and 584 Log K_{ow} between -3.7 and 5.49	Published article indicated good reliability and accuracy.	https://www.aiha.org/get-involved/VolunteerGroups/Pages/Exposure-Assessment-Strategies-Committee.aspx	Excel	Model accessible to EASC volunteer group (members only)		AIHA's Exposure Assessment Strategies Committee (EASC) and the Dermal Project Team (DPT) in collaboration with Wil ten Berge	english									NvG
**IndusChem Fate**	IndusChemFate	Different Targets	inhalation, dermal, oral		generic PBTK-model, applicable for volatile and semi-volatile chemicals	Screening level		respiratory system not described in detail, therefore less suitable for particles seems to be limited to chemicals with a short half-life in the body		http://cefic-lri.org/toolbox/induschemfate/	Excel-spreadsheet	free		CEFIC LRI	english							2.0	2011	NvG

**IVIVE-Fish BCF**	In Vitro-In Vivo Extrapolation: Fish Bioconcentration Factor (IVIVE – Fish BCF) Model	fish		organic chemcials						https://arnotresearch.com/IVIVE/	online	free	active	ARC	english									
**IV-MBM**	In Vitro Mass Balance Model (IV-MBM)	in vitro data	in vitro experimental set up	organic chemicals			true concentration in vitro			https://arnotresearch.com/IV-MBM/	online and excel	free	active	ARC	english									
**PK-SIM**	PK-SIM	Consumer	oral							http://www.open-systems-pharmacology.org/		free			english									NvG
**PLETHEM**	Plethem: Population Life-course exposure to health effects model	Consumer	various							CRAN (https://cran.r-project.org/web/packages/plethem/index.html) or Github	R package	free			englisch				open source hub for models					NvG
**SimCyp**	SimCyp	Consumer	PBPK	drugs																				
